# Structure Formation in the Wetting Layer of a Carbonyl‐Functionalized Ionic Liquid on Au(111): How to Control the Functional Group?

**DOI:** 10.1002/cphc.202500229

**Published:** 2025-07-02

**Authors:** Lukas Knörr, Hanna Bühlmeyer, Julien Steffen, Simon Trzeciak, Jonas Hauner, Dirk Zahn, Andreas Görling, Jörg Libuda

**Affiliations:** ^1^ Interface Research and Catalysis, ECRC Friedrich‐Alexander‐Universität Erlangen‐Nürnberg Egerlandstraße 3 91058 Erlangen Germany; ^2^ Chair of Theoretical Chemistry Friedrich‐Alexander‐Universität Erlangen‐Nürnberg Egerlandstraße 3 91058 Erlangen Germany; ^3^ Computer Chemistry Center, CCC Friedrich‐Alexander‐Universität Erlangen‐Nürnberg Nägelsbachstraße 25 91052 Erlangen Germany

**Keywords:** Au(111), density functional theory, infrared reflection absorption spectroscopy, ionic liquids, molecular dynamics, scanning tunneling microscopy, solid catalysts with ionic liquid layers

## Abstract

Coating heterogeneous catalysts with ionic liquids (ILs), a strategy known as ‘solid catalysts with ionic liquid layers’, can fine‐tune catalytic selectivity. Introducing functional groups into ILs enhances their interaction with reactants, but precise control over their positioning is crucial. The structural formation in the IL wetting layer of the carbonyl‐functionalized IL [5‐oxo‐C_6_C_1_Im][NTf_2_] on Au(111) is investigated using infrared reflection absorption spectroscopy and scanning tunneling microscopy under ultrahigh vacuum conditions, supported by density functional theory and molecular dynamics simulations. At low temperatures (<130 K), the IL forms disordered islands, which coalesce into ordered films near ambient temperature. At low coverage, the IL adopts flat, space‐demanding adsorption geometries. Upon forming a closed film, adsorption shifts to more compact configurations, with the carbonyl group tilting toward the vacuum while the ring remains surface‐bound. Deposition at 300 K forms crystalline structures in the sub‐monolayer regime, where the cation side chain can either stand upright or lie flat depending on the coverage. The IL remains thermally stable and desorbs completely at 500 K without decomposition. These findings highlight how IL coverage and deposition conditions tune functional group orientation at the catalyst interface, optimizing SCILL performance.

## Graphical abstract

Understanding how functional groups influence ionic liquid adsorption is key to optimizing SCILL catalyst interfaces. Using IRAS, STM, DFT, and MD, we studied [5‐oxo‐C6C1Im][NTf_2_] on Au(111). Temperature and coverage determine whether the cations lie flat in space‐demanding motifs or adopt compact geometries with carbonyl groups tilting towards vacuum. Ordered films form near 260 K, and the IL desorbs intact at 500 K.

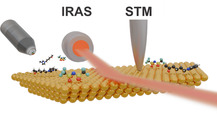

## 1 Introduction

In recent years, coatings by ionic liquids (**IL**) have emerged as a novel approach to improve heterogeneous catalysts.^[^ ^1^, ^2^, ^3^ ^]^ ILs are salts with melting points below 100 °C and negligible vapor pressure.^[^ ^2^, ^4^ ^]^ A promising application of ILs are solid catalysts with ionic liquid layers (**SCILL**).^[^ ^3^, ^5^, ^6^, ^7^, ^8^, ^9^, ^10^, ^11^, ^12^, ^13^, ^14^, ^15^ ^]^ In these applications, conventional supported catalysts are coated with thin IL films.^[^ ^1^, ^2^ ^]^ These IL films can significantly improve the catalytic characteristics, such as the selectivity, of common heterogenous catalysts by blocking unwanted consecutive and side reactions. Furthermore, by tailoring the size, shape, and functional groups of the ILs the efficiency of a catalyst toward certain reactions can be enhanced even more.^[^ ^4^ ^]^

An important reaction is the heterogeneous catalyzed hydrogenation of carbonyl compounds. However, the activation of the stable C=O bond poses a challenge for hydrogenation.^[^ ^16^ ^]^ Research indicates that the conversion of the keto group to the corresponding enol form may enable hydrogenation of the CO group at low temperature.^[^ ^17^, ^18^, ^19^ ^]^ This assumption is based on the fact that the hydrogenation of the C=C group is more favorable by ≈35 kJ mol^−1^ compared to the C=O group.^[^ ^20^ ^]^ However, the enol species tends to revert to the more stable ketone species.^[^ ^21^ ^]^ Recent studies on Pt(111) have shown that, under certain reaction conditions, adsorbed acetophenone forms ketone‐enol dimers.^[^ ^22^, ^23^ ^]^ In light of this, the application of a ketone‐functionalized IL as SCILL might further improve the stability of the enol species.

**Figure 1:**
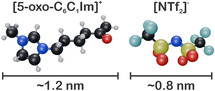
Ball model of [5‐oxo‐C_6_C_1_Im][NTf_2_], showing the approximate lengths of the two ions.

As a preliminary investigation, the adsorption, interaction, and thermal stability of a ketone‐functionalized IL on metal single crystals will be examined. In our previous research, we investigated the carbonyl‐functionalized IL 1‐(5‐oxo‐hexyl)‐3‐methyl‐imidazolium‐bis‐(trifluormethylsulfonyl)‐imid [5‐oxo‐C_6_C_1_Im][NTf_2_] on Pt(111).^[^ ^5^ ^]^ We found that the IL starts to decompose at around 260 K on this substrate. In this work, we extend our investigation to 1‐(5‐oxo‐hexyl)‐3‐methyl‐imidazolium‐bis‐(trifluormethylsulfonyl)‐imid [5‐oxo‐C_6_C_1_Im][NTf_2_] on Au(111). **Figure** 1 shows the molecular structure and dimensions of the IL. To this end, we employ a combination of infrared reflection absorption spectroscopy (**IRAS**) and scanning tunneling microscopy (**STM**) under ultrahigh vacuum (**UHV**) conditions, complemented by density functional theory (**DFT**) and molecular dynamics (**MD**) simulations.

## 2 Results and Discussion

### 2.1 IRAS: Adsorption and Thermal Stability of [5‐oxo‐C_6_C_1_Im][NTf_2_] Films Deposited at 130 K

**Figure 2:**
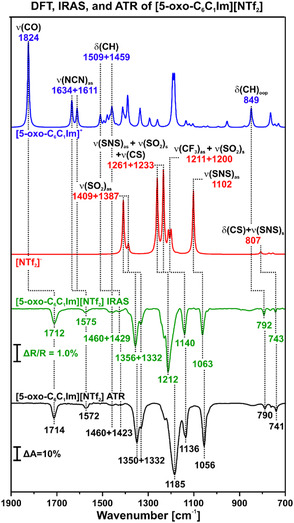
DFT (blue and red), multilayer IRA spectra (green) and ATR (black) of [5–oxo–C_6_C_1_Im][NTf_2_]. The multilayer IRA spectrum was performed at 130 K. The most intense signals are labeled and assigned according to DFT calculations. (PBE0^[^ ^49^ ^]^ ‐D4^[^ ^50^ ^]^ /def2‐TZVPP^[^ ^51^, ^52^ ^]^ orca5^[^ ^53^, ^54^ ^]^).

We deposited [5‐oxo‐C_6_C_1_Im][NTf_2_] on Au(111) by physical vapor deposition (PVD) in UHV. In Figure S1, Supporting Information, we show the time‐resolved IRA spectra for the growth of a multilayer film (≈4 ML). Additionally, we performed an Attenuated Total Reflectance infrared (ATR‐IR) spectrum under ambient conditions as a reference and complemented it with DFT‐calculated spectra. In **Figure** **2**, we compare the resulting IR spectra, revealing a similarity between the measured and the calculated data. From this observation, we conclude that the IL evaporates under UHV conditions without decomposition. In **Table** **1** we present the assignment of the DFT, ATR, and IRAS signals. We based the assignment of the IR signals on the literature values of similar ILs^[^ ^7^, ^9^, ^10^ ^]^ and DFT‐calculations.

**Table 1:** Assignment of the DFT, IRAS, and ATR signals of [5‐oxo‐C_6_C_1_Im][NTf_2_].

DFT [cm^−1^]	IRAS [cm^−1^]	ATR [cm^−1^]	Assignment
1824	1712	1714	ν(CO)
1634 + 1611	1575	1572	ν(NCN)_as_
1509 + 1459	1460 + 1429	1460 + 1423	δ(CH)
1409 + 1387	1356 + 1332	1350 + 1332	ν(SO2)_as_
1233	1212	1185	ν(SNS)_as_ + ν(SO2)_s_ + ν(CS)
1211 + 1200	1140	1136	ν(CF3)_as_
1102	1063	1056	ν(SNS)_as_
849	792	790	δ(CH)_oop_
807	743	741	δ(CS) + ν(SNS)_s_

In Figure S2, Supporting Information, we present the temperature‐programed IRA spectra for the multilayer film. We observe that the multilayer desorbs until 385 K. This result aligns with the desorption behavior of the [5‐oxo‐C_6_C_1_Im][NTf_2_] multilayer on Pt(111), which we also observed in this temperature region.^[^ ^5^ ^]^ Additionally, we detect remaining signals up to ≈500 K. These signals correspond to the residual monolayer, which remains stable longer compared to the multilayer due to its interactions with the Au(111) surface.

**Figure 3:**
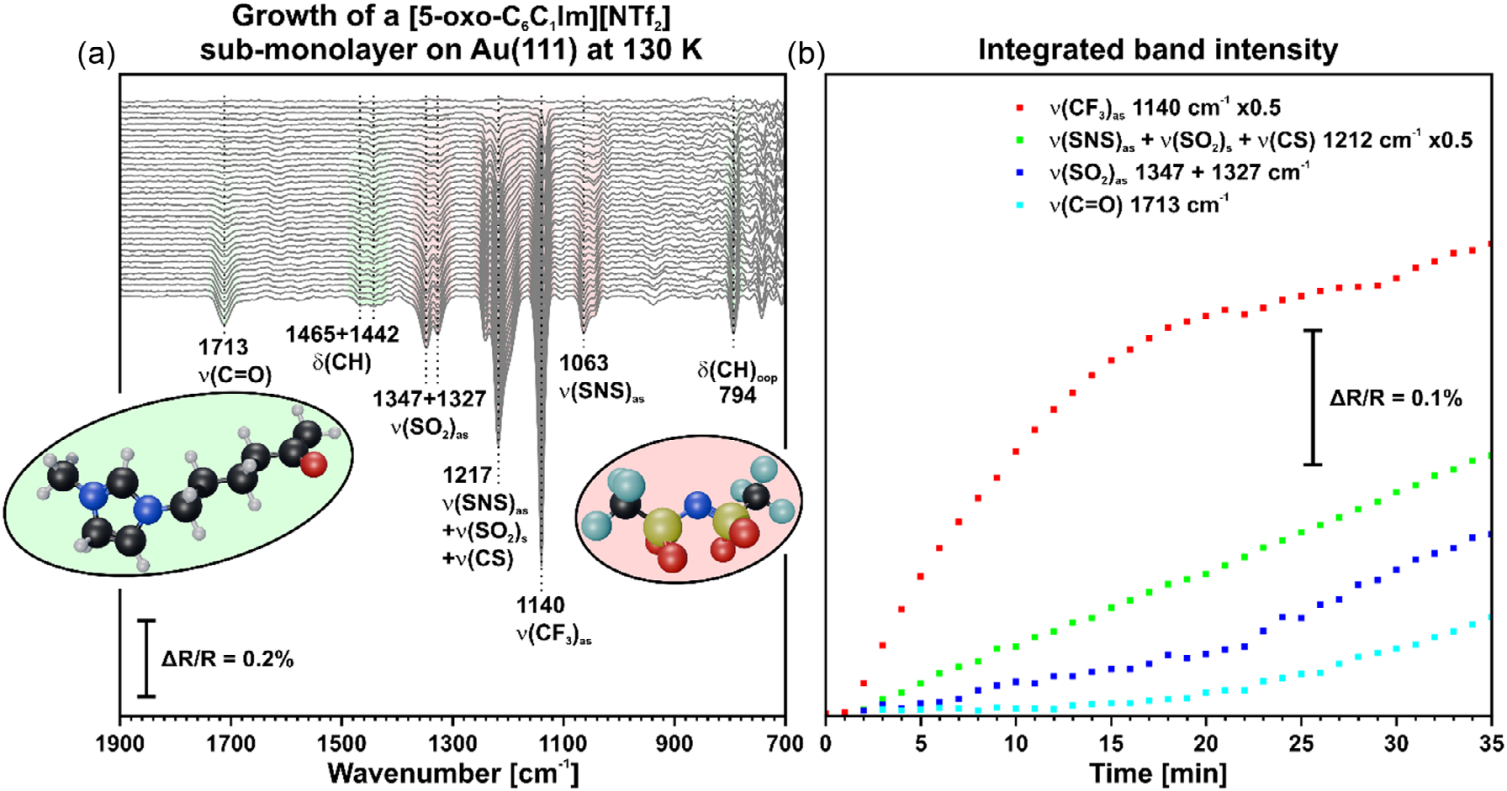
a) In situ IRA spectra recorded during the PVD of [5‐oxo‐C_6_C_1_Im][NTf_2_] on Au(111) at 130 K. The spectra show the growth of a sub‐monolayer of the IL. b) Integrated band intensity of the most prominent signals.

**Figure 4:**
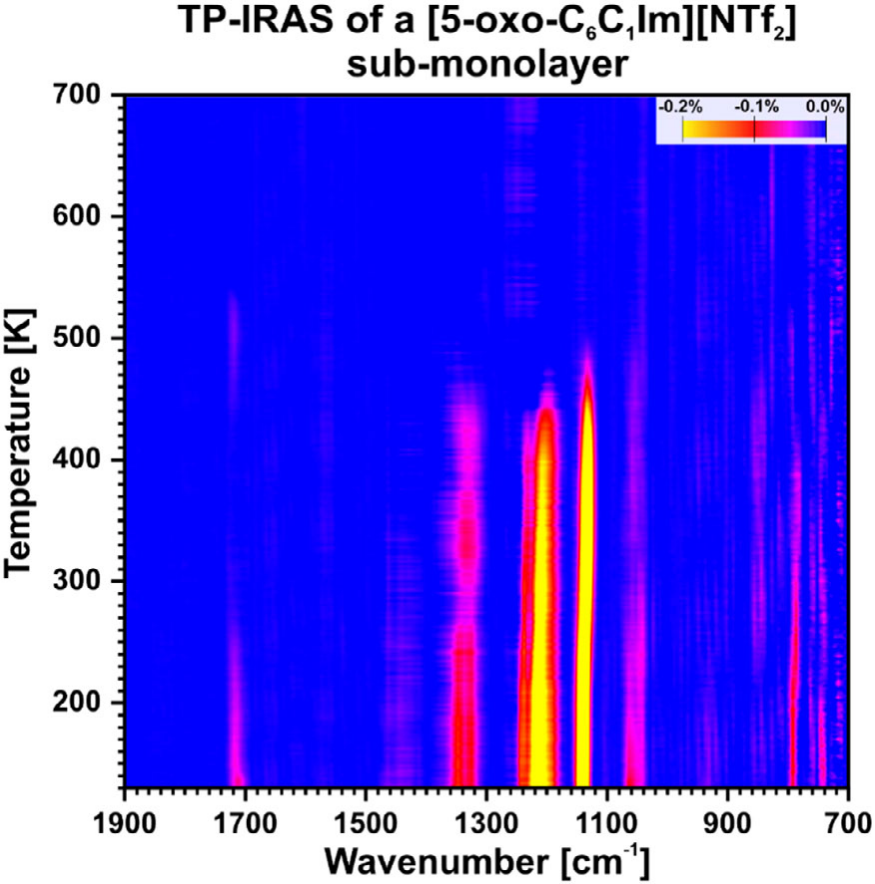
TP‐IRAS of a [5‐oxo‐C_6_C_1_Im][NTf_2_] sub‐monolayer on Au(111) from 130 to 700 K, plotted as color plot.

**Figure 5:**
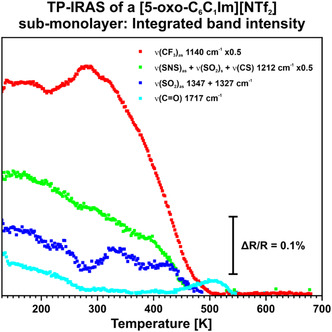
TP‐IRAS of a [5‐oxo‐C_6_C_1_Im][NTf_2_] sub‐monolayer on Au(111) from 130 to 700 K, featuring the integrated band intensity of the most significant signals. The sub‐monolayer was deposited at 130 K.

Next, we studied the growth of a [5‐oxo‐C_6_C_1_Im][NTf_2_] sub‐monolayer on Au(111). To ensure that we deposited a sub‐monolayer, we performed an adsorption experiment at 400 K beforehand. At this temperature, no multilayer can form, and only a monolayer remains at the surface. We present the corresponding spectra in Figure S3, Supporting Information. For the sub‐monolayer, we selected the coverage such that the intensity of the signals was below that of a monolayer. During the deposition of the IL, we recorded time‐resolved IR spectra at 130 K. (see **Figure** **3**a) All spectra are referenced to the clean Au(111) surface. Upon deposition of the IL, two bands appear at 1217 and 1134 cm^−1^. We assign the first signal to the coupled vibration of ν(SNS)_as_, ν(SO_2_)_s_, and ν(CS). The second band corresponds to the ν(CF_3_)_as_ vibration. With increasing coverage, new bands appear at 1442, 1347, 1327, 1063, and 794 cm^−1^. We attribute these bands to the δ(CH), ν(SO_2_)_as_, ν(SNS)_as_, and δ(CH)_oop_ vibrations. Additionally, the band at 1134 cm^−1^ shifts to 1140 cm^−1^ as the coverage increases. At even higher coverage, a new signal occurs at 1713 cm^−1^. We assign this band to the ν(CO) stretching mode of the cation. Considering the metal surface selection rule (MSSR), only vibrations with a dynamic dipole moment parallel to the surface normal can be excited in the IRAS experiment on Au.^[^ ^24^ ^]^ Therefore, the delayed appearance of the carbonyl vibration indicates that the carbonyl group of the cation is oriented parallel to the Au(111) surface at low coverage. This observation suggests that the alkyl chain of the cation is thus anchored on the surface due to dispersion forces. As the coverage increases, the alkyl chain undergoes a detachment from the surface and orients itself toward the vacuum. Therefore, additional adsorption sites open up on the surface. As a result, the parallel alignment of the carbonyl to the surface is lost, and the ν(CO) mode gains intensity. In Figure 3b, we show the band heights of the most prominent signals as a function of the deposition time. In this representation, it is more apparent that the ν(CO) signal appears at larger coverages only. Uhl et al.^[^ ^14^ ^]^ investigated the adsorption motif of [C_8_C_1_Im][NTf_2_] on Au(111) with STM. Cremer et al.^[^ ^12^ ^]^ investigated the same IL on Au(111) with X‐ray photoelectron spectroscopy. In both studies, the authors concluded from their data that at low sub‐monolayer coverage, the cation is adsorbed with the alkyl chain lying flat on the interface. Near the monolayer‐region, they observed that the alkyl chain starts to reorient toward the vacuum.^[^ ^12^, ^14^ ^]^ These findings are in line with the results of our experiments with the functionalized ionic liquid. Furthermore, we observe in Figure 3b that the growth rate of the signals at 1063 cm^−1^, attributed to the ν(SNS)_as_ vibration, and the bands at 1347 and 1327 cm^−1^, ascribed to the ν(SO_2_)_as_ vibration, exhibit an increase concurrent with the observation of the ν(CO) signal. Hohner et al.^[^ ^10^ ^]^ investigated a similar ionic liquid, namely [C_2_C_1_Im][NTf_2_] on Pt(111). They observed that the [NTf_2_]^−^ anion adsorbs predominantly in a 2–1 orientation, with one SO_2_ group binding over both O atoms and one SO_2_ group binding over a single O atom. Additionally, they observed the appearance of a 2–0 orientation as the monolayer region is reached (i.e., one SO_2_ group detached from the surface). Therefore, we attribute the increase in intensity of the ν(SNS)_as_ and ν(SO_2_)_as_ signals to a reorientation of the anion to an upright adsorption motif, which appears upon reaching the monolayer region. Subsequently, we conducted a study on the thermal stability and desorption behavior of the sub‐monolayer film using temperature‐programed IRAS. (Refer to the experimental section for further details.) In **Figure** **4**, we present the related data between 130 and 700 K as 3D color plot. In **Figure** **5** we show the integrated band intensity for the most significant signals. At 130 K, we observe similar bands to those during the deposition experiment. (see Figure 3 and Table 1) Between 130 and 143 K, we observe a shift of the ν(CO) signal from 1713 to 1717 cm^−1^ as well as a fast drop of intensity of the ν(CO) signal. Additionally, starting from 143 K, we observe a further decrease of the ν(CO) signal until it vanishes at ≈300 K. We note that the other signals also feature a slight decrease in intensity starting from 143 K. However, they remain visible in the spectrum. Furthermore, we observe that the ν(CF_3_)_as_ signal at 1140 cm^−1^ increases between 250 and 300 K. Between 300 and 500 K we note a strong decrease of the ν(CF_3_)_as_ signal. At ≈500 K, we observe that all signals vanish. We attribute the initial shift of the ν(CO) signal from 1713 to 1717 cm^−1^ as well as the decrease of intensity starting from 143 K to a reorientation effect. This indicates that the IL at 130 K initially adsorbs in an amorphous structure. Considering the MSSR, the decrease of the ν(CO) signals indicates a reorientation to a more crystalline structure. In this case, the CO group lies parallel to the Au(111) surface. Talwar et al.^[^ ^25^ ^]^ investigated a nitrile‐functionalized IL on Au(111). They observed decomposition of the IL between 375 and 400 K. Additionally, in a recent study, we investigated [5‐oxo‐C_6_C_1_Im][NTf_2_] on Pt(111). In this case, we observed decomposition of the IL in both IRAS and STM starting at around 260 K.^[^ ^5^ ^]^ Conversely, we do not observe indications of decomposition in the temperature‐programmed infrared (TP‐IRA) spectra. Therefore, we assume that keto‐functionalized ILs are more stable on Au(111) as compared to nitrile‐functionalized ILs.

### 2.2 IRAS: Adsorption and Thermal Stability of [5‐oxo‐C_6_C_1_Im][NTf_2_] Films Deposited at 300 K

In the preceding section, we concluded that the CO group of the cation reorients to a parallel adsorption motif between 143 and 300 K. In order to investigate this phenomenon with respect to the influence of the deposition temperature, we deposited [5‐oxo‐C_6_C_1_Im][NTf_2_] on Au(111) by PVD in UHV at 300 K. In Figure S4, Supporting Information, we show the time‐resolved IRA spectra for the growth of a sub‐monolayer of [5‐oxo‐C_6_C_1_Im][NTf_2_] on Au(111) at 300 K. It is important to note that we deposited the same coverage as for the previous experiment at 130 K. We detect the same signals as for the deposition at 130 K. However, we identify a shift of the ν(CO) signal from 1713 to 1720 cm^−1^. We also note a shift in the coupled vibration of ν(SNS)_as_, ν(SO_2_)_s_, and ν(CS) from 1217 cm^−1^ to 1211 cm^−1^, and in the ν(CF_3_)_as_ signal from 1140 cm^−1^ to 1137 cm^−1^. The largest shift we observe occurs in the ν(SNS)_as_ signal from 1063 cm^−1^ to 1048 cm^−1^. Since we previously observed that the IL sub‐monolayer reorients at ≈143 K, we suggest that the shift in wavenumbers arises from a more ordered IL adsorption motif on the Au(111) surface.

**Figure 6:**
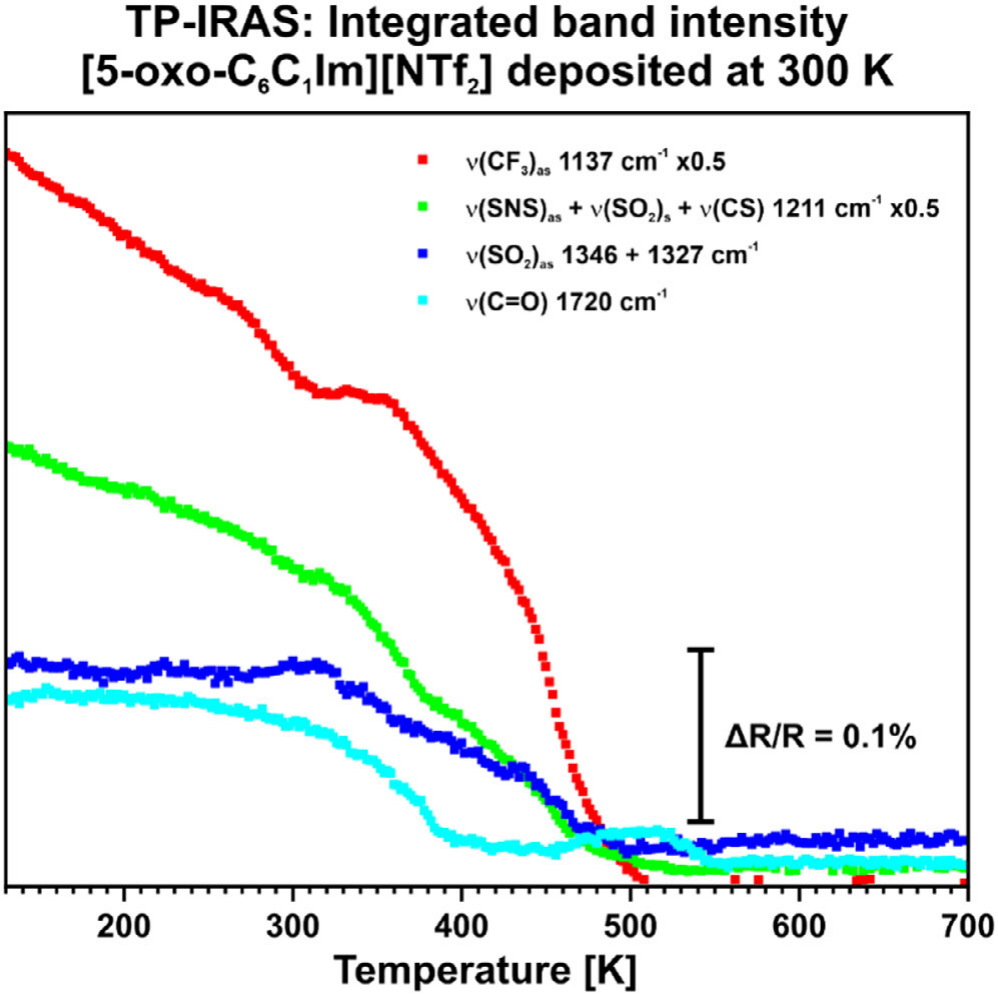
TP‐IRAS of a [5‐oxo‐C_6_C_1_Im][NTf_2_] sub‐monolayer on Au(111) from 130 to 700 K, featuring the integrated band intensity of the most significant signals. The sub‐monolayer was deposited at 300 K.

Subsequently, we investigated the thermal stability of this sub‐monolayer film. To compare the results to the sub‐monolayer film deposited at 130 K, we cooled the system down to 130 K. From this point, we used the same heating ramp of 2 K min^−1^ as for the experiments conducted previously. In Figure S5, Supporting Information, we present the related data between 130 and 700 K as 3D color plot. In **Figure** **6**, we show the integrated band intensity for the most significant signals. At 130 K, we observe the same signals and the same signal ratio as in the deposition experiment. (see Figure S4, Supporting Information) Upon heating, we note that the ν(CO) signal is stable until ≈250 K and the ν(SO_2_)_as_ signals remains stable until about 325 K. However, the ν(CF_3_)_as_ and the compound signal at 1211 cm^−1^ slowly decrease until ≈350 K. Upon reaching this temperature, the signal intensity decreases faster until it vanishes at 500 K. Starting from 250 K, we observe a decrease of the ν(CO) signal until it disappears at ≈400 K. In the previous section we observe the decline of the ν(CO) signal starting from 143 K. However, in the sub‐monolayer film, prepared at 300 K, this signal remains stable until 250 K, at which point it commences a decrease. We attribute this decrease to the reorientation of the cation to a flat adsorption motif. Uhl et al.^[^ ^13^ ^]^ investigated a pyrrolidinium‐based IL on Au(111), in which the cation exhibits a long aliphatic chain. They found that their IL forms a 2D glass phase and a crystalline phase. They found that the glass phase melts at 113 K while the corresponding crystalline phase melts at 173 k. Furthermore, they found that domains with a long‐range order show even higher melting points, up to 225 K. In consideration of these findings, we assume that the [5‐oxo‐C_6_C_1_Im][NTf_2_] IL forms a crystalline structure on Au(111) with the chains of the cation pointing to the vacuum. This crystalline structure thus exhibits enhanced stability at elevated temperatures in comparison to the amorphous structure observed in the previous section.

To investigate the influence of the IL coverage further, we performed a similar experiment at lower IL coverage. In Figure S6, Supporting Information, we show the time‐resolved IRA spectra for the growth of [5‐oxo‐C_6_C_1_Im][NTf_2_] on Au(111) at 300 K with lower coverage as compared to the previous experiment. We observe the same signals as for both previous experiments conducted at 130 and 300 K. However, we note that the ν(CO) signal is missing. Additionally, we observe that the ν(SO_2_)_as_ signal comprises only one band at 1329 cm^−1^ instead of two bands at 1346 and 1327 cm^−1^ as observed in the previous deposition experiments. This indicates a different adsorption motif of the sub‐monolayer on the Au(111) surface compared to the previous experiments. With regard to the MSSR, the missing ν(CO) signal indicates a parallel CO group and, thus, a flat orientation of the cation on the Au(111) surface.

**Figure 7:**
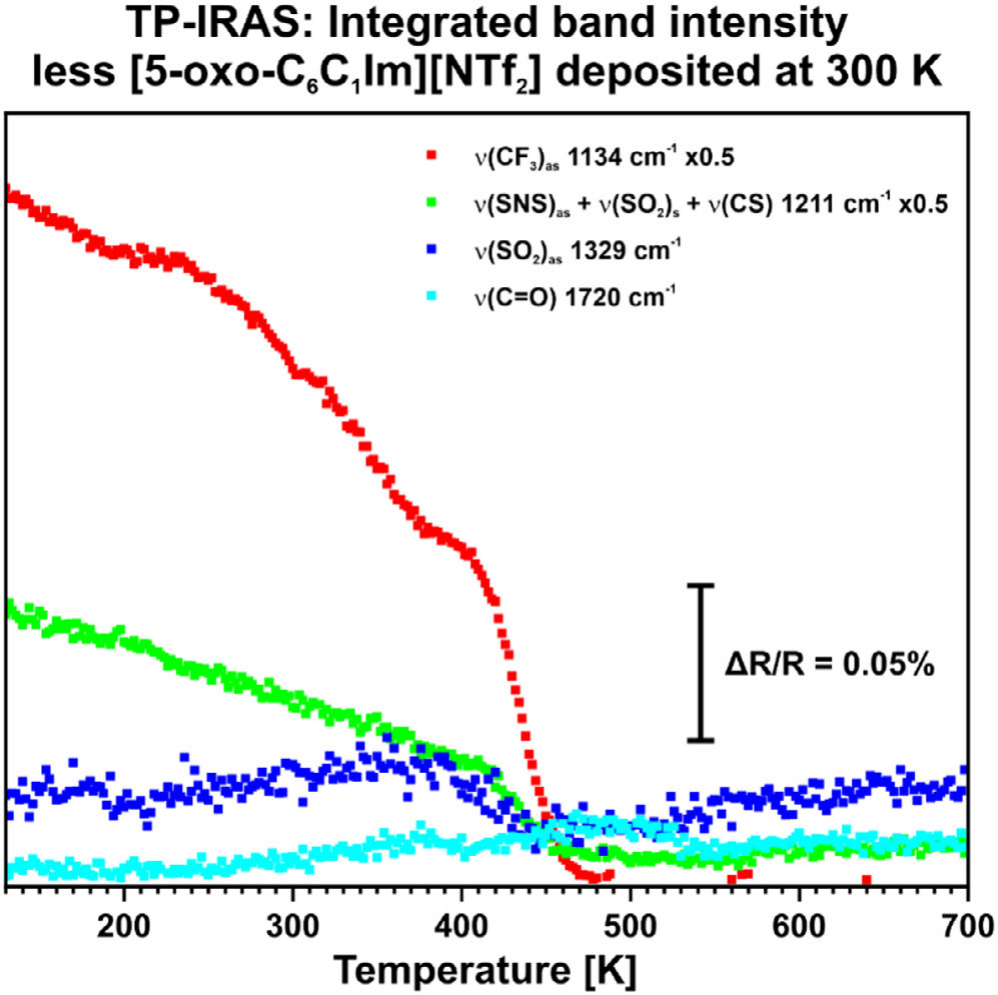
TP‐IRAS of a [5‐oxo‐C_6_C_1_Im][NTf_2_] sub‐monolayer on Au(111) from 130 to 700 K, featuring the integrated band intensity of the most significant signals. The sub‐monolayer was deposited at 300 K. Less IL was deposited on the surface compared to Figure 5.

Afterwards, we investigated the thermal stability of the IL film on Au(111). To this end, we cooled the sample down to 130 K to compare the results with the previous experiments. In Figure S7, Supporting Information, we present the related data between 130 and 700 K as 3D color plot. In **Figure** **7** we show the integrated band intensity for the most significant signals. Upon heating, we observe that the ν(SO_2_)_as_ signal remains stable until ≈400 K. The ν(CF_3_)_as_ and the compound signal at 1211 cm^−1^ decrease until ≈400 K. Upon reaching this temperature, all signals decrease faster until they vanish at 500 K. We identify a signal at 1720 cm^−1^ at ≈300 K, which increases until 500 K. However, in the 3D color plot it is evident that there are fragments in the spectrum around this wavenumber until 700 K. **(**Figure S7, Supporting Information**)** We assume that the increase of the signal at 1720 cm^−1^ results from background fragments due to temperature change of the sample. Considering the MSSR, the absence of the ν(CO) signal indicates a parallel alignment of the keto group in respect to the Au(111) surface. In accordance with this finding, we assume that the aliphatic chain of the cation lies on the Au(111) surface in a parallel manner due to dispersion forces. Furthermore, we assume that the cation exhibits a tendency to adsorb in a flat configuration, provided that sufficient adsorption sites are available. This configuration is characterized by a flat‐lying imidazolium ring and a parallel aligned keto group with respect to the Au(111) surface. Conversely, if the IL adsorbs in a confined space with limited adsorption sites, as we observed in the previous experiment where we dosed a higher amount of IL on the surface, the aliphatic chain of the cation exhibits the tendency to disconnect from the surface and points toward the vacuum.

### 2.3 STM: Adsorption and Thermal Stability of [5‐oxo‐C6C1Im][NTf2] at Different Temperatures

**Figure 8:**
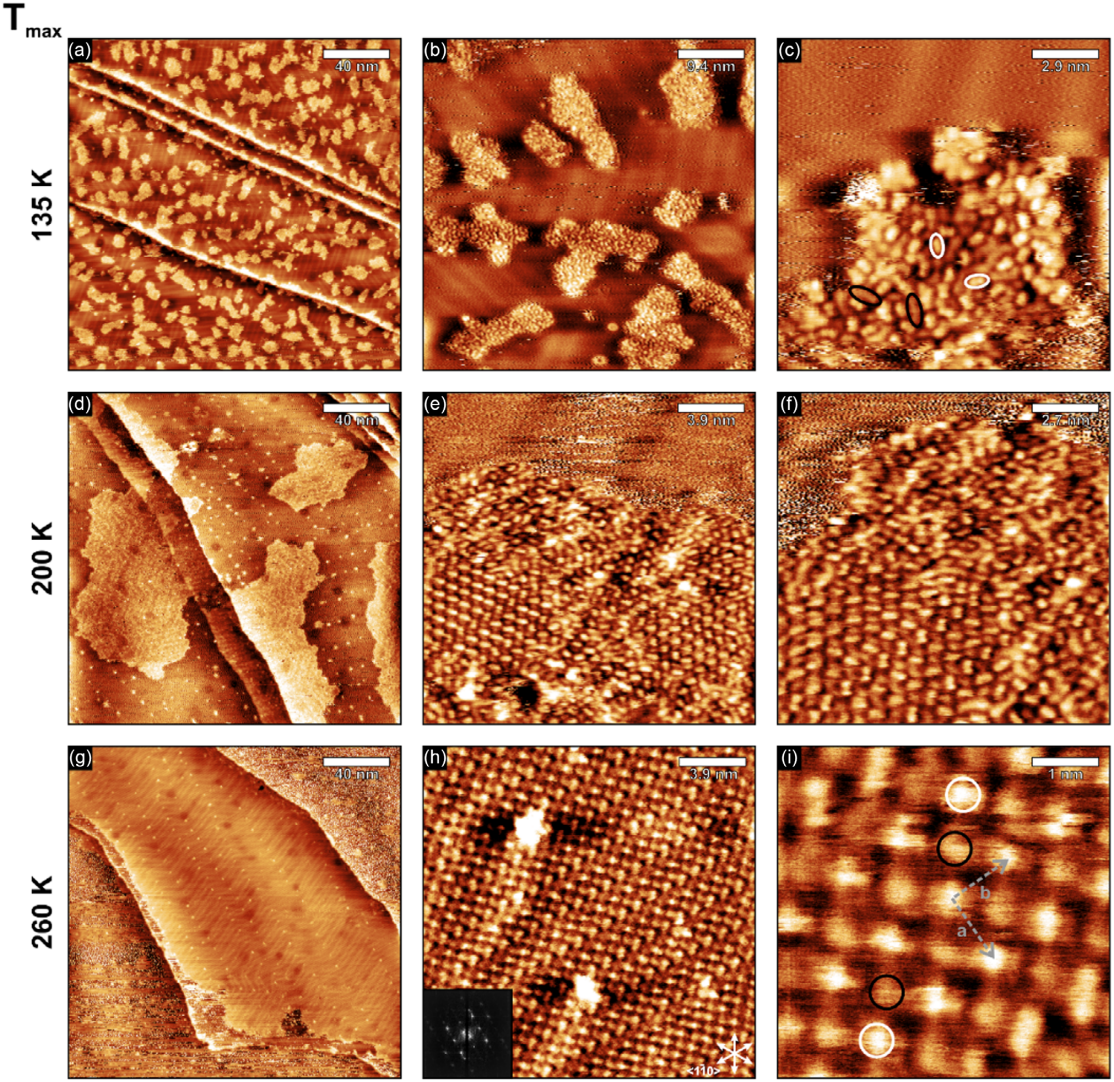
STM images of [5‐oxo‐C_6_C_1_Im][NTf_2_] films on Au(111); a–c) measured after deposition at 135 K, d–f) after annealing to 200 K, and g–i) after annealing to 260 K. All STM images were measured at <130 K. See Table S1, Supporting Information, for detailed preparation and scanning parameters.

In addition to the IRAS experiments, we investigated [5‐oxo‐C_6_C_1_Im][NTf_2_] on Au(111) by STM to obtain detailed information on the local adsorption and structure of the IL. We prepared thin films of [5‐oxo‐C_6_C_1_Im][NTf_2_] on Au(111) by PVD of the IL at temperatures between 120 K and 135 K under UHV conditions. Subsequently, we transferred the sample to the STM, which was cooled to temperatures below 130 K with liquid nitrogen to prevent temperature increase. For measurements, the IL is frozen, and thus, surface diffusion is inhibited. At the same time, the scanner unit was counter‐heated to RT. Furthermore, to investigate the thermal stability of [5‐oxo‐C_6_C_1_Im][NTf_2_], we performed temperature‐dependent STM experiments by heating the sample to a desired temperature, keeping it at this temperature, and then cooling it back down to the measuring temperature below 130 K. In **Figure** **8** we show the thermal evolution of [5‐oxo‐C_6_C_1_Im][NTf_2_] at different temperatures. The images are recorded directly after deposition of [5‐oxo‐C_6_C_1_Im][NTf_2_] and after heating to 200 K and 260 K, respectively. For each temperature, one representative overview image and two close‐ups are shown.

Immediately after preparation, the surface is partially covered by 2D IL islands (Figure 8a–c). The herringbone structure of the Au(111) surface is clearly resolved between the IL islands. The IL islands appear in various shapes and sizes but do not form a continuous film at these temperature (Figure 8a–c). As we already observed for [C_2_C_1_Im][OTf] on Au(111),^[^ ^8^ ^]^ there is a preferred adsorption site on Au(111).^[^ ^15^, ^26^ ^]^ The IL forms domains at both the elbows of the herringbone reconstruction and the step edges, with these sites acting as nucleation centers for stronger adsorption. At higher magnification, we are able to image the internal structure of the islands. In Figure 8b,c, we observe mainly two types of protrusions. Namely, bright, elliptical protrusions as well as less bright, longer protrusions. In Figure 8c, we marked the bright and the less bright features by white and black ovals, respectively. We observed similar features for [5‐oxo‐C_6_C_1_Im][NTf_2_] on Pt(111) in our previous publication.^[^ ^5^ ^]^ The assignment of the two different features in the STM images is based on the adsorption geometry derived from IRAS (see IRAS part) and previous studies on comparable systems.^[^ ^5^, ^10^, ^12^, ^27^ ^]^ We assign the bright, elliptical features to the [NTf_2_]^−^ anion, which binds via its SO_2_ group in a parallel adsorption motif to the Au(111) surface. The less bright, longer feature is attributed to the [5‐oxo‐C_6_C_1_Im]^+^ cation, which lies flat on the surface. This finding is in accordance with the results we observed for [5‐oxo‐C_6_C_1_Im][NTf_2_] on Pt(111).^[^ ^5^ ^]^ It is noteworthy that at this temperature we observe no long‐range order, which we also found for [C_2_C_1_Im][OTf] on Au(111).^[^ ^8^ ^]^ Thus, at a temperature of 120 K to 135 K, the molecules do not rearrange to a 2D crystalline film. This indicates limited surface mobility at this temperature. Individual ions or ion pairs are sufficiently mobile to form small islands at 130 K, but not extended films. However, mobility within and along the edges of the existing islands appears slower, which contributes to their amorphous structure.

Annealing to 200 K results in larger IL islands (Figure 8d–f) and, thus, in a change in wetting behavior. We also observed this behavior for [C_2_C_1_Im][OTf] on Au(111).^[^ ^11^ ^]^ As we observed before, the islands predominantly appear at the step edges and grow between the herringbones of the Au(111) reconstruction. In this context, the elbows function as nucleation centers. Further expansion of the islands indicates a higher mobility at this temperature in comparison to temperatures below 200 K. Additionally, some islands show two different structures at the same time. On the one hand, we observe an amorphous structure consisting of multiple different features. On the other hand, a crystalline area with a hexagonal structure is formed within the amorphous islands.

Further heating to 260 K results in greater changes in the morphology of the IL islands on the Au(111) surface (Figure 8g–i). The IL now appears as a continuous film that covers large parts of the terraces and even entire steps. Certain islands exhibit an amorphous structure. However, we observe films with a well‐defined crystalline structure over large areas. In this case, the arrangement of the features is well defined. The protrusions appear congruent with the directions of the Au(111) reconstruction and form a checkerboard pattern. The primitive unit cell (marked in Figure 8i) is spanned by vector $\left|\vec{a}\right|=1.04\;\mathrm{nm}$ and vector $\left|\vec{b}\right|=1.00\;\mathrm{nm}$, with an angle of ≈90° in between. The crystalline structure with azimuthal order is also illustrated in the FT of the STM image (Figure 8h). In Figure 8h,i we observe two types of protrusions within the crystalline structure. We marked the bright and the less bright features by white and black circles, respectively (Figure 8i). A numerical analysis of the two protrusions yielded a 1:1 ratio. Straightforward, the bright protrusion corresponds to one ion, while the less bright one represents the other ion. In this scenario, anions and cations are arranged adjacent to one another and making direct contact with the surface, as it was previously observed for different ILs on Au(111).^[^ ^8^, ^11^, ^12^, ^15^, ^26^, ^28^ ^]^ We assign the bright feature to the [5‐oxo‐C_6_C_1_Im]^+^ cation, which is adsorbed with its side chain pointing toward the vacuum, and the less bright one to the [NTf_2_]^−^ anion, similar to Uhl et al.^[^ ^13^, ^14^, ^15^ ^]^ Both the bright and the less bright features appear mainly as roundish protrusions, however, some lines show elliptical protrusions. We assume that these features represent the same ions but in a different adsorption motif (see DFT). From IRAS, DFT, MD, and literature^[^ ^7^, ^10^, ^12^, ^27^ ^]^ we assume that the [5‐oxo‐C_6_C_1_Im]^+^ cation and [NTf_2_]^−^ anion can adsorb in different adsorption motifs depending on the available space on the surface. At low coverages, the ions adsorb in a space‐demanding motif on the surface, whereas at coverages close to one monolayer, the ions reorient to a more upright, space‐saving adsorption motif. We assume that confined space is the driving force for crystallization with space‐saving adsorption motifs. The higher temperature leads to a mobile IL, which then accumulate in larger islands and continuous films. In this case, herringbones, terraces, and amorphous structures can act as a barrier and create a confined area for the IL adsorbates. This results in local areas with IL coverages similar to a monolayer. Within this monolayer, the ions change their adsorption motifs from space‐demanding to space‐saving, even though the flat‐lying motif is more stable than the more upright standing motifs. Consequently, the space‐saving motifs allow more ions to be placed on the same surface area. This squeezed monolayer film is more stable than a layer in a flat‐lying configuration with a second layer on top, which was also found for other ILs.^[^ ^10^, ^29^ ^]^

### 2.4 MD Simulations: Adsorption Motif and Film Formation Energy of [5‐oxo‐C_6_C_1_Im][NTf_2_]

**Figure 9:**
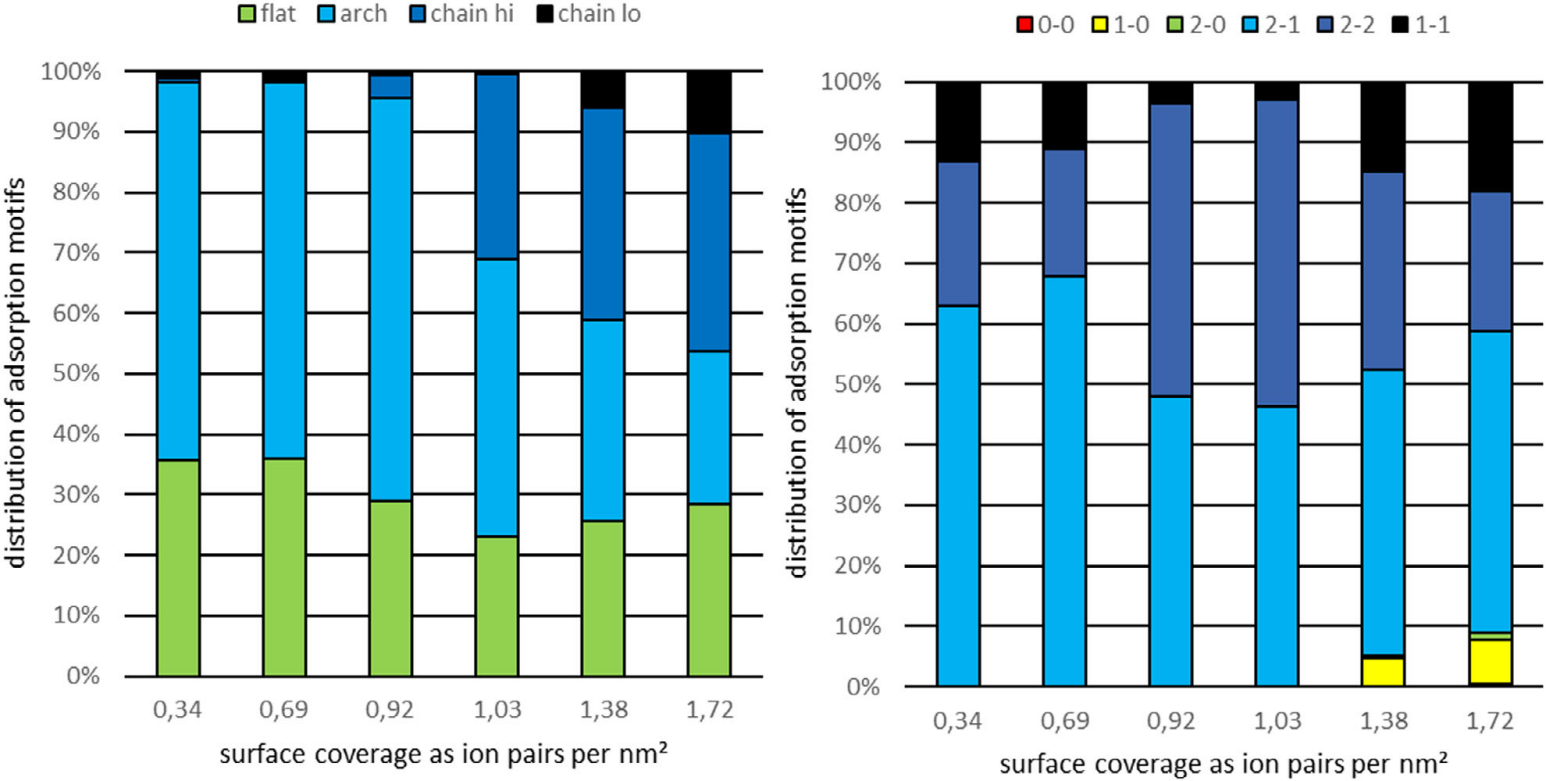
Conformer analysis of the ionic liquid [5‐oxo‐C_6_C_1_Im][NTf_2_] on Au(111) at 150 K as a function of surface coverage (IP/nm^2^). The analysis for the [5‐oxo‐C_6_C_1_Im]^+^ cation is shown on the left, and for the [NTf_2_]^−^ anion on the right.

To support the experimentally obtained findings with theoretical results, we conducted additional MD simulations to elucidate the adsorption motifs present in the examined system and their variation with surface coverage at different temperatures. In order to identify the dominant adsorption motifs under the specific conditions, we performed analyses for both the [5‐oxo‐C_6_C_1_Im]^+^ cation and the [NTf_2_]^−^ anion. In **Figure** **9** we show a conformer analysis at 150 K.

Similar to the experimental section, we observe that the [5‐oxo‐C_6_C_1_Im]^+^ cation adsorbs flat on the gold surface due to the aromatic nature of the imidazole‐based ring. The small size of the methyl substituent limits detailed analysis, so it is generally considered to adsorb flat. In contrast, the carboxy‐functionalized hexyl chain can adopt various configurations on the metal surface, and we analyzed it specifically to identify its adsorption motifs. From this, the motifs are labeled as “*flat*”, “*arch*”, “*chain hi*” and “*chain lo*”. We characterize the “*flat*” motif by the substituent lying flat on the surface, with the keto group–in time averaged manner–aligned parallel to the gold surface. In the “*arch*” motif, the alkyl chain bends toward the vacuum, while the keto group remains on the gold surface. We define the “*chain hi*” motif in a way that the keto group detaches from the gold surface, while the chain points toward the vacuum. This adsorption motif is the most space‐saving option of those four. Conversely, the “*chain lo*” motif occurs exclusively in multilayers, with the substituent directed toward the gold surface and the ring positioned unstructured above the monolayer.

For the [NTf_2_]^−^ anion, previous studies^[^ ^10^ ^]^ identified both space‐saving and space‐demanding adsorption motifs. The motifs differ in the number of oxygen atoms that interact with the gold surface. The space‐demanding motifs include “*1‐1*”, “*2‐2*”, and “*2‐1*”. The space‐saving motifs are “*2‐0*” and “*1‐0*”. Note that the notation “*i‐j*” represents the number of oxygen atoms in contact with the gold surface.

In Figure 9 we show both the cation (left) and the anion (right). As long as a complete monolayer does not exist (<0.9 IP nm^−^^2^), the ions predominantly adopt space‐demanding adsorption motifs. Once a complete monolayer is formed (≈0.9 IP nm^−^^2^), a transition occurs from space‐demanding to space‐saving adsorption motifs. For the cation, the proportion of “*flat*” and “*arch*” motifs decreases in favor of “*chain hi*”, resulting in a more compact surface structure. In contrast, for the anion, the “*1‐1*” and “*2‐1*” motifs first shift toward the “*2‐2*” motif until parts of the anion shift toward the *“1‐1”* and *“1‐0”* configuration.

**Figure 10:**
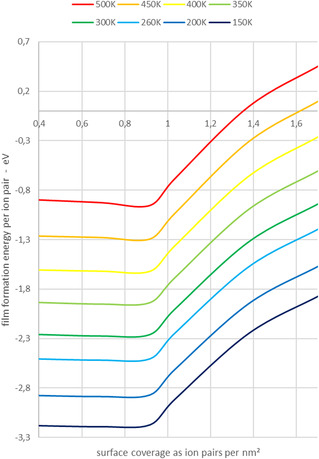
Film formation energy per ion pair as a function of surface coverage and temperature.

The identification of the closed monolayer at ≈0.9 IP/nm^2^ is further supported by the film formation energy, as shown in **Figure** **10**. In the sub‐monolayer region, the incorporation energy for ion pairs remains nearly constant. Note, we define 1 ML of the IL as a closed layer of chemisorbed ion pairs, that is, the maximum number of species that are in direct contact with the surface. From the formation of the monolayer, which represents a turning point, it decreases continuously with the number of ion pairs. Additionally, for each surface coverage, the film formation energy decreases linearly with increasing temperature.

**Figure 11:**
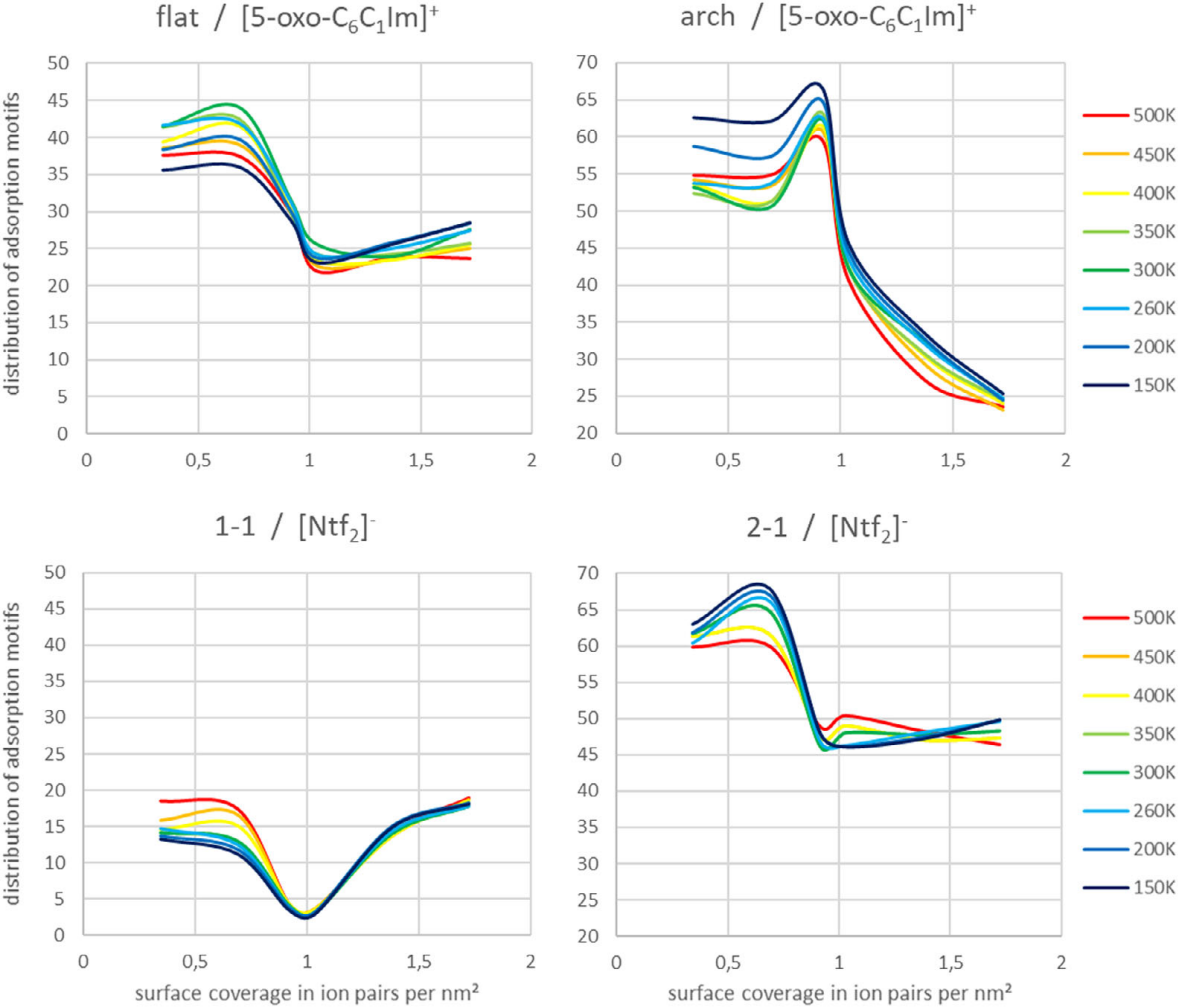
Conformer analysis of the ionic liquid [5‐oxo‐C6C1Im][NTf2] on Au(111) as a function of surface coverage (IP/nm^2^) and temperature. The analysis for the [5‐oxo‐C6C1Im]^+^ cation is shown on the top–on the left for the “flat” and on the right for the “arch” motif. The analysis for the [NTf2]^−^ anion is shown on the bottom–on the left for the “1‐1” and on the right for the “2‐1” motif.

Considering the temperature dependance of the adsorption motifs of both ions, only the motifs “*flat*”, “*arch*”, “*1‐1*”, and “*2‐1*” exhibit significant temperature dependance, within the statistical accuracy of a standard deviation of less than 3%. Refer to **Figure** **11**.

As shown in Figure 9, the ions adopt space‐saving adsorption motifs during the transition from the sub‐monolayer to the monolayer, occurring uniformly across all considered temperatures. Additionally, the proportion of relatively more space‐saving motifs, such as “*arch*” and “*2‐1*” (Figure 11, right), decreases with rising temperature in favor of the more space‐demanding motifs, “*flat*” and “*1‐1*” (Figure 11, left). We attribute this phenomenon to the increased mobility of the ions at higher temperatures, leading to relaxation within the films formed. With increasing temperature, IL–IL interactions become increasingly important and drive sub‐monolayer film reorganization in favor of arrangements that are characteristic of the monolayer. We also observed this effect in the IRAS data in the previous section.

### 2.5 DFT: Adsorption Motif and Geometry of [5‐oxo‐C_6_C_1_Im][NTf_2_]

In order to identify preferred adsorption motifs and adsorption patterns of [5‐oxo‐C_6_C_1_Im][NTf_2_] on Au(111), we performed DFT simulations. In the first step, we optimized 20 different dimer adsorption motifs on a 7 × 7 Au(111) surface unit cell. We placed the [NTf_2_]^−^ anion in cis and trans configurations and the [5–oxo–C_6_C_1_Im]^+^ cation in both flat and upright configurations (where the side chain points toward the vacuum). Additionally, we set up the ions with different orientations and center‐of‐mass locations with respect to the surface. In Figure S8 and Table S2, Supporting Information, we show six representative optimized adsorbed dimers, together with their total energy and adsorption energy. Note that the first three and the last one feature the highest (negative) adsorption energy out of the tested adsorption motifs. Adsorption motifs four and five are representative examples of other adsorption patterns with high energies. Therefore, we observe the most stable adsorption motif with the side chain of the [5–oxo–C_6_C_1_Im]^+^ cation lying flat or in an arched manner on the Au(111) surface. It is important to note that the keto group is invariably oriented in a parallel manner in respect to the Au(111) surface. We observe the [NTf_2_]^−^ anion also lying flat. However, we note that the adsorption energy mainly depends on the orientation of the cation, as the energy is very similar if one, two, or all four oxygen atoms of the anion are directed toward the surface. The calculations are in agreement with our IRAS and STM observations, as we observed that the flat adsorption motifs of the IL are most common at low coverages where the IL is not confined in its adsorption area.

**Figure 12:**
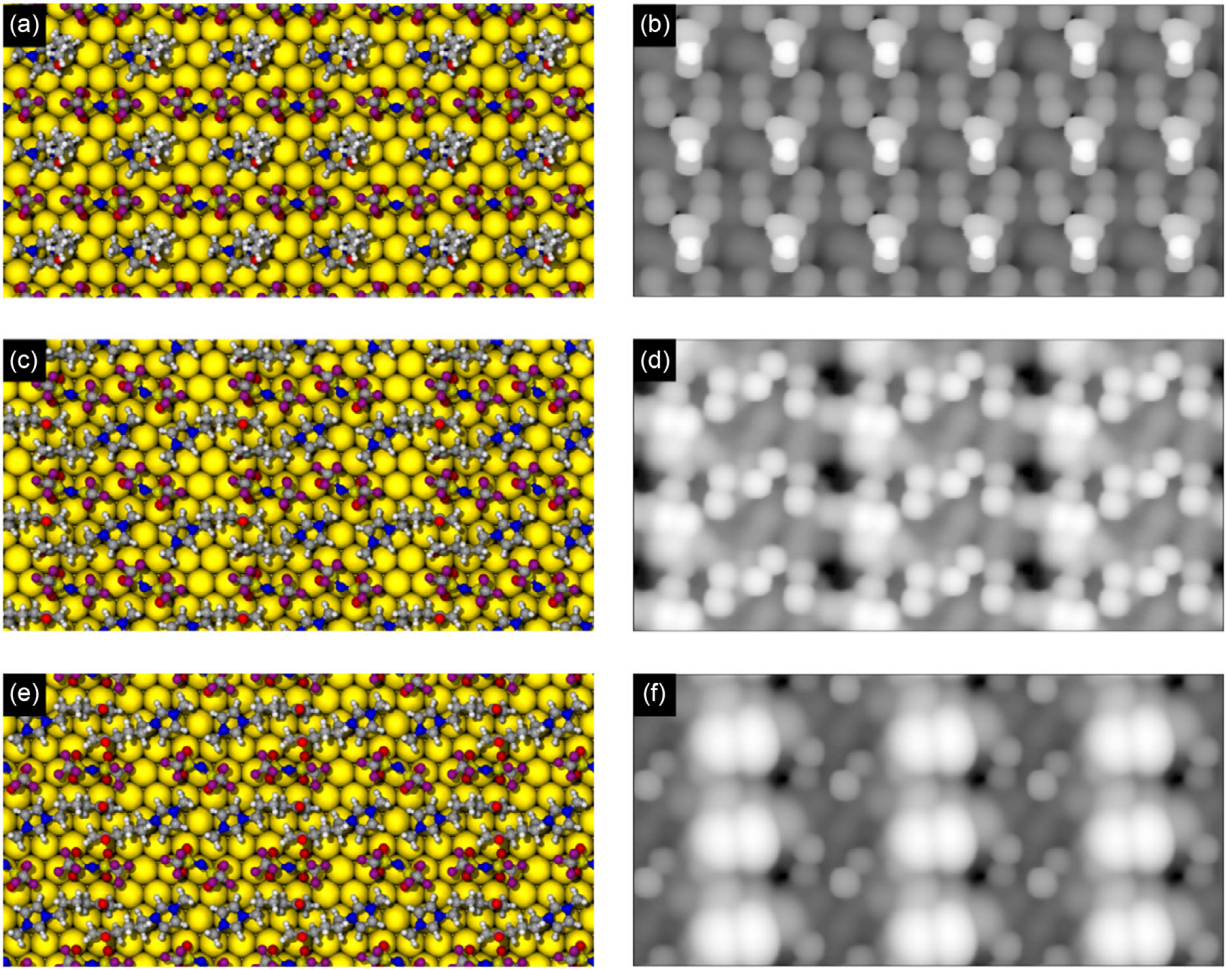
DFT‐calculated adsorption structures and corresponding simulated STM images of different adsorption motifs. The left side presents top‐view optimized structures, while the right side displays their corresponding simulated STM images. In (a) and (b), the cation adopts an upright configuration with its side chain pointing toward the vacuum. In contrast, (c) and (d), as well as (e) and (f), depict adsorption motifs where the cation lies flat on the Au(111) surface, showcasing different spatial arrangements.

Next, we optimized surface adsorption patterns to simulate the measured STM images. We designed the initial distributions of the IL molecules on the Au(111) surface such as to reproduce the distribution of the ions extracted from the experimental STM images (see unit cell Figure 8i). Note that we focus our discussion mainly on the [5‐oxo‐C_6_C_1_Im]^+^ cation, as a detailed study on the different adsorption motifs of the [NTf_2_]^−^ anion on Pt(111) can be found in literature.^[^ ^10^ ^]^ For simplification, in this study, we placed the [NTf_2_]^−^ anions always in their *cis*‐conformation. We show the final adsorption geometries and the STM images of the adsorption patterns in **Figure** **12**. In the previous section we noted that a flat‐lying cation with an arched side chain has the highest adsorption energy. However, if the adsorption area is confined, we observe an upright cation in the IRAS and STM part. Therefore, we simulated STM images not only for an arched cation but also for an adsorption motif with an upright side chain. In Figure 12a, we observe the side chain of the [5‐oxo‐C_6_C_1_Im]^+^ cation pointing toward the vacuum. The simulated STM image (Figure 12b) shows regular protrusions, which originate from the side chains of the cations. These bright protrusions alternate with less bright protrusions originating from the [NTf_2_]^−^ anions, forming a checkerboard structure. In Figure 12c and Figure 12e, we observe that the side chain of the [5‐oxo‐C_6_C_1_Im]^+^ cation adsorbs in an arched position on the surface (Figure S8, Supporting Information Structure 6). In the simulated STM images of this motif (Figure 12d and Figure 12f), we also observe a checkerboard‐like structure. Both the calculated geometries and the simulated STM images are in good agreement with the measured STM images (Figure 8h,i). However, the pattern with the side chain of the [5‐oxo‐C_6_C_1_Im]^+^ cation pointing toward the vacuum appears to be more consistent with the experimental data and lends support to our interpretation of the STM and IRAS data. It is important to note that in the experimental studies, the [NTf_2_]^−^ anion most likely adsorbs in an upright adsorption motif rather than flat at higher coverages. We assume that this is also the reason why the protrusions for the same ions do not always appear identical in the measured STM images (Figure 8h,i). In most cases, the anions appear as a single, roundish feature rather than two (compared to DFT simulation). However, the adsorption motif of the [NTf_2_]^−^ anion has not been considered in these DFT simulations. Furthermore, the feature attributed to the [5‐oxo‐C_6_C_1_Im]^+^ cation appears as either a single, roundish feature or as an elliptical feature consisting of two roundish ones. Thus, we propose that the crystalline structure consists of anions and cations alternating on the surface, with the ions adsorbing mainly in two different adsorption motifs. Namely, as upright or arched cations and upright or flat anions, with one adsorption motif always appearing in a complete line (see Figure 8h–i).

## 3 Summary and Conclusion

In this work, we studied the adsorption and structure formation of a thin film of [5–oxo–C_6_C_1_Im][NTf_2_] on Au(111) at different temperatures. To this end, we combined MD simulations and DFT calculations with experimental data obtained by time‐resolved and temperature‐programed IRAS experiments as well as STM experiments under UHV conditions. We conclude the following: 1) At temperatures below 130 K, [5‐oxo‐C_6_C_1_Im][NTf_2_] forms isolated 2D islands with no long‐range order. As the temperature rises to ≈140 K, the IL begins to reorient and combine into larger islands. At 260 K, continuous IL films cover large parts of the terraces. Films with a well‐defined crystalline structure over large areas are observed. 2) The IL preferentially adsorbs at the step edges and within the herringbone reconstruction of the Au(111) surface, with these areas acting as nucleation centers for island formation. 3) The IL predominantly adsorbs in space‐demanding adsorption motifs, if enough adsorption sites are available. Thus, the [5‐oxo‐C_6_C_1_Im]^+^ cation adsorbs in flat or arched motifs with the imidazole ring and the carbonyl group aligned parallel to the Au(111) surface. 4) Upon reaching a closed film, the IL adopts space‐saving adsorption motifs. The carbonyl group detaches from the surface and orients toward the vacuum, while the imidazole ring remains firmly attached to the surface. 5) When deposited at 300 K, the IL forms different crystalline structures in the sub‐monolayer regime, with the side chain of the cation either standing upright or lying flat, depending on the dose and packing density. 6) The IL fully desorbs at 500 K without undergoing decomposition.

## 4 Experimental Section

4.1

4.1.1

##### IRAS

The in situ sample preparation and IRAS experiments were conducted in an UHV setup with a base pressure of 1.0 × 10^−10^ mbar, comprising two sub‐chambers. The first chamber was used for sample cleaning and included an ion gun (Specs IQE 11/35), a quartz crystal microbalance (QCM, Inficon SQM‐160), a low‐energy electron diffraction (LEED) optics (Specs ErLEED 150), a gas dosing system, and a quadrupole mass spectrometer (QMS, Blazers Quadstar 422). The second sub‐chamber included a remote‐controlled effusive beam source, several custom‐built Knudsen cells, and a QMS (Hiden Analytical Hal 3 F). To record the IR spectra, a vacuum fourier‐transform infrared spectrometer (Bruker VERTEX 80v) was employed, equipped with a liquid nitrogen‐cooled mercury cadmium telluride detector. The Au(111) single crystal (MaTeck) was cleaned by multiple cycles of Ar^+^ sputtering (Ar, Linde, >99.9999%; 1.2 keV, 8.5 × 10^−5^ mbar, 30 min) and annealing (950 K in UHV; 15 min). The cleanliness of the single crystal was checked with LEED. [5‐oxo‐C_6_C_1_Im][NTf_2_] was evaporated from a glass reservoir using a home‐built Knudsen cell. The evaporator was isolated from the main chamber by a gate valve, which was connected to a separate high vacuum line. Prior to the deposition, the evaporator was heated for 20 min with the gate valve closed. The deposition was monitored by simultaneous acquisition of IR spectra (1 min/spectrum, spectral resolution of 4 cm^−1^). Each spectrum was then referenced to the spectrum of the clean surface recorded before deposition. For the TP‐IRAS experiment, the temperature of the sample was ramped with a rate of 2 K min^−1^, while simultaneously recording IRA spectra (1 min/spectrum). The attenuation of the IR signals with increasing temperature was corrected for by normalization of the spectra as previously described by Xu et al.^[^ ^30^ ^]^

##### STM

The sample preparation and STM measurements were performed in a SPECS UHV system with a base pressure <1.0 × 10^−10^ mbar consisting of two sub‐chambers. The sample was cleaned and prepared in the preparation chamber. The STM measurements were carried out in the analysis chamber with an SPM 150 Aarhus microscope. In previous publications, a detailed description of the setup could be found.^[^ ^8^ ^]^

The Au(111) single crystal (MaTeck) was cleaned by Ar^+^ sputtering (Ar, Linde, >99.9999%; 1.0 keV, 2 × 10^−5^ mbar, 30 min), and subsequent annealing (950 K in UHV; 10 min). The cleanliness of the single crystal was checked with STM. The Au(111) sample was cooled to 180–200 K before the IL was evaporated from a home‐built Knudsen cell in form of a glass reservoir loaded with [5‐oxo‐C_6_C_1_Im][NTf_2_]. The evaporator was separated from the main chamber by a gate valve and pumped via a separate high vacuum line. For deposition, the evaporator was preheated for 60 min. After preparation, the sample was quickly transferred to the analysis chamber. For low temperature measurements, the STM was cooled with liquid nitrogen while the scanner unit was counter‐heated to room temperature (RT). For temperature‐dependent experiments, the STM was heated to the desired temperature along with the sample and maintained at this temperature for several hours. Afterward, the STM and the sample were cooled back down to the measurement temperature. The temperature was monitored using a thermocouple attached to the STM block. Since the STM metal block was in thermal contact with the sample via the sample stage, it was assumed that the STM block and the sample were in thermal equilibrium and at the same temperature. The STM metal block was heated by passing current through two Zener diodes mounted on the block, with a maximum current of 100 mA and a voltage of 70 V applied to each diode. Cooling was achieved by circulating liquid nitrogen through the metal block. The STM images were recorded in constant‐current mode with a tungsten tip and a SPECS Kolibri sensor, using a bias voltage of ≈1.0–1.6 V applied to the sample and a tunneling current of ≈200–900 pA. The images were treated and evaluated with Gwyddion software.

##### DFT

Periodic DFT calculations of the system were carried out with the VASP code, where a plane wave basis set for the description of the valence electrons is used combined with the projector augmented wave method for the representation of atomic cores.^[^ ^31^, ^32^, ^33^ ^]^ The kinetic energy cutoff was set to 500 eV, exchange correlation effects were treated with the PBE functional.^[^ ^34^ ^]^ Since generalized gradient approximation functionals such as PBE are known to inadequately describe dispersion interactions, we employed the widely used empirical DFT D3 correction scheme with Becke–Johnson damping.^[^ ^35^, ^36^ ^]^ Electronic states were smeared with the Methfessel–Paxton scheme (first order) and a broadening of 0.15 eV.^[^ ^37^ ^]^ In gas‐phase calculations, Gaussian smearing with a broadening of 0.04 eV was used. The Au(111) surface was modeled by surface slabs of four layers. The cell size was determined by an initial optimization of a gold‐bulk cell containing 4 atoms (20 × 20 × 20 Gamma‐containing k‐point mesh), the distances between the atoms (2.933 Å) were taken as initial positions for the surface slabs. The cell vectors and the bottom two metal atom layers were held fixed during the further calculations. The [5‐oxo‐C_1_C_6_Im]^+^ cation and the [NTf_2_]^−^ anion were also optimized separately in the gas phase, where a cubic box with axes lengths of 25 Å was used. The charges of the ions were produced by changing the total number of electrons in the system with the Number of ELECTrons (NELECT) keyword combined with applying a constant background countercharge. For the optimization of (approximately) isolated [5‐oxo‐C_6_C_1_Im][NTf_2_] dimers adsorbed on the Au(111) surface, the initial placements of the dimers on the surface slabs (7 × 7 hexagonal slabs) were done with the new Python script build_adsorbates.py from the utils4VASP Github repository [https://github.com/Trebonius91/utils4VASP]. The geometries were optimized until all cartesian force components were below 0.02 eV/Å.

IL adsorption patterns on Au(111) were modeled using two IL pairs placed on a surface slab of 7 × 4 orthogonal Au(111) unit cells (a = 20.5326 Å, b = 10.1610 Å), with different locations of the anions and cations and different initial configurations of the cations, resulting in 18 different initial structures. Brillouin zone sampling for isolated dimers on the 7 × 7 Au(111) slab was performed using a Γ‐centered 2 × 2 × 1 k‐point mesh, while a 2 × 4 × 1 mesh was applied for the 7 × 4 Au(111) cell. For gas‐phase molecule species, only the Γ‐point was sampled. We have demonstrated in previous studies^[^ ^8^, ^11^, ^38^ ^]^ that the chosen k‐point meshes were suitable for a good description of the electronic structure while keeping the calculation effort manageable. Since isolated gas‐phase species were simulated without any interactions between the periodic images, using only the Γ‐point for them was the usual choice. The geometries were relaxed until all Cartesian force components were below 0.02 eV/Å. Although the herringbone reconstruction significantly influences the structure of the Au(111) surface, its large periodic pattern makes it impractical for modeling with periodic DFT. Future modeling of reconstructed Au(111) surfaces will likely require machine‐learned potentials.^[^ ^39^ ^]^ However, these approaches struggle to provide a stable and accurate description of the ionic liquid and its various adsorption patterns. The expected fitting error in machine‐learned potentials will likely exceed the energetic impact of surface reconstruction on IL adsorption. For the relaxed IL adsorption patterns, scanning tunneling microscope images were simulated with the eval_stm program from the utils4VASP repository on Github. The Tersoff–Hamann approach in the constant‐current mode has been used, the charge density was integrated from the Fermi level until 1 eV below it. A modeled tip broadened by a Gaussian of width of 0.3 Å was lowered in each grid point until a charge density of 0.1 1/Å^3^ was reached. The resulting distribution of z‐values was then plotted as simulated STM image.

##### MD Simulation

The simulation software used in this study, the Large‐scale Atomic/Molecular Massively Parallel Simulator, with the code version dated June 23, 2022, was employed to analyze the film formation behavior of the functionalized ionic liquid [5‐oxo‐C_6_C_1_Im][NTf_2_] on a Au(111) surface, as well as its structural properties. The timestep used for integration was 1.0 fs. For temperature regulation of the systems under consideration, the Nose‐Hoover thermostat with a relaxation time constant of 0.5 ps was applied.^[^ ^40^ ^]^ van der Waals interactions were described using the Lennard‐Jones potential with a cutoff of 12 Å. Long‐range electrostatics were handled with the particle‐particle particle‐mesh method^[^ ^41^ ^]^ and a 2D Ewald slab correction.^[^ ^42^ ^]^ Partial charges for the IL were generated via quantum chemical calculations of electrostatic potentials using Gaussian 09 (revision D.01) at the HF/6‐31 G* level. Subsequently, the partial charges were determined following the “*restricted electrostatic potential*” method using the Antechamber tool from the Amber 22 package.^[^ ^43^ ^]^ Interaction parameters for the ionic liquid were obtained from the general amber force field II 2.11 suite, as published in the Amber 22 package.^[^ ^44^ ^]^ Interaction parameters for Au atoms were taken from the study by Heinz et al.^[^ ^44^ ^]^ All partial charges and interaction parameters are available in the Supporting Information.

In contrast to previous studies,^[^ ^8^, ^11^ ^]^ a reconstructed Au(111) surface, known as the “*Herringbone Reconstruction*”, was used instead of an ideal Au(111) surface for the MD simulations. For this, a relaxed slab with dimensions of 31.71 nm (x) and 7.63 nm (y) was taken from the study by Li et al.^[^ ^39^ ^]^ was initially tripled along the y‐axis to create a surface area of 725.46 nm^2^, ensuring that the periodic structure of the herringbones is fully represented within the simulation box at least once, without being truncated at the edges. This size is sufficient to eliminate finite size effects influencing the structuring of the ionic liquid on the surface and to allow the study of more than a single elbow of the “*Herringbone Reconstruction*”, thereby better understanding its impact on the ionic liquid's structure. Furthermore, the original slab was taken from the stydy by Li et al.^[^ ^39^ ^]^ contained 32 explicit atomic layers, which were reduced to the top six layers. This reduction was based on the fact that the interaction of the ionic liquid with the metal surface occurs solely via Lennard‐Jones potentials within a range of 12 Å, rendering deeper gold atoms unnecessary. Prior studies^[^ ^7^, ^8^, ^11^, ^45^ ^]^ also indicated that a corelaxing metal surface has no impact on the structuring of IL. Hence, the prepared slab was held fixed during the simulations. The resulting gold slab has a thickness of 1.2 nm. Perpendicular to the surface normal, an 8.7 nm vacuum terminated by a repulsive wall was introduced to prevent molecules from leaving the 2D periodic system. Film generation followed procedures similar to those in previous studies.^[^ ^8^, ^11^ ^]^ In parallel simulations, 250, 500, 666, 750, 1000, and 1250 ion pairs were used to create corresponding surface coverages of 0.34, 0.69, 0.92, 1.03, 1.38, and 1.72 ion pairs per nm^2^. These ion pairs were artificially placed in a vacuum and preequilibrated at 1000 K. Subsequently, simulated annealing was employed with a cooling rate of 10 K/ns, which had proven effective in earlier works,^[^ ^7^, ^8^, ^11^, ^45^, ^46^, ^47^, ^48^ ^]^ to reduce the temperature to 150 K. The system was equilibrated at this temperature for 5 ns before statistically relevant sampling over an additional 5 ns. Starting from 150 K, temperature‐dependent screening of the systems was conducted. The systems were heated in 50 K increments at a heating rate of 5 K/ns, equilibrated for 5 ns, and then sampled for another 5 ns.

## Conflict of Interest

The authors declare no conflict of interest.

## Supporting information

Supplementary Material

## Data Availability

The data that support the findings of this study are openly available in Zenodo at https://doi.org/10.5281/zenodo.14882132, reference number 14882132.
