# Supplementary material for: Structure Formation in the Wetting Layer of a Carbonyl‐Functionalized Ionic Liquid on Au(111): How to Control the Functional Group?
Source: Chemphyschem. 2025 Jul 2;26(16):e202500229. doi: 10.1002/cphc.202500229 (PMC12388164; doi:10.1002/cphc.202500229)
Supplement: Supplementary file 1 — Supplementary Material [file CPHC-26-e202500229-s001.pdf]

# Supporting Information

# Structure Formation in the Wetting Layer of a Carbonyl-Functionalized Ionic Liquid on Au(111): How to Control the Functional Group?

Lukas Knörr[1§], Hanna Bühlmeyer[1§], Julien Steffen[2], Simon Trzeciak[3], Jonas Hauner[1], Dirk Zahn[3], Andreas Görling[2], Jörg Libuda[1*]

[1] *Interface Research and Catalysis, ECRC, Friedrich-Alexander-Universität Erlangen-Nürnberg, Egerlandstraße 3, 91058 Erlangen, Germany*

[2] *Chair of Theoretical Chemistry, Friedrich-Alexander-Universität Erlangen-Nürnberg, Egerlandstraße 3, 91058 Erlangen, Germany*

[3] *Computer Chemistry Center, CCC, Friedrich-Alexander-Universität Erlangen-Nürnberg, Nägelsbachstraße 25, 91052 Erlangen, Germany*

§ These authors contributed equally

*corresponding author: Jörg Libuda, joerg.libuda@fau.de

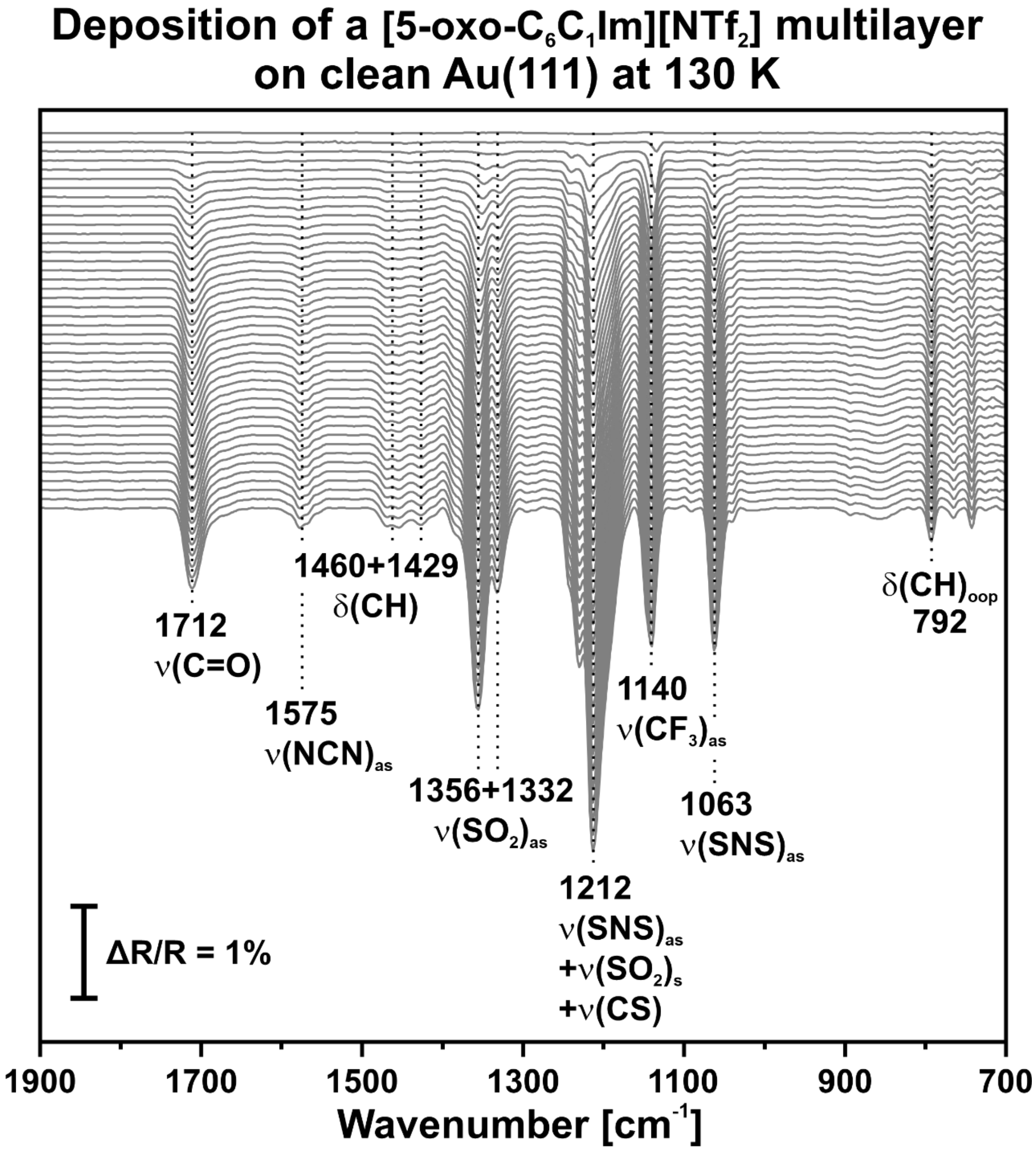


**Figure S1:** In-situ IRA spectra recorded during PVD of [5-oxo-$C_6C_1$Im][$NTf_2$] on Au(111) at 130 K. The spectra show the growth of a multilayer of the IL.

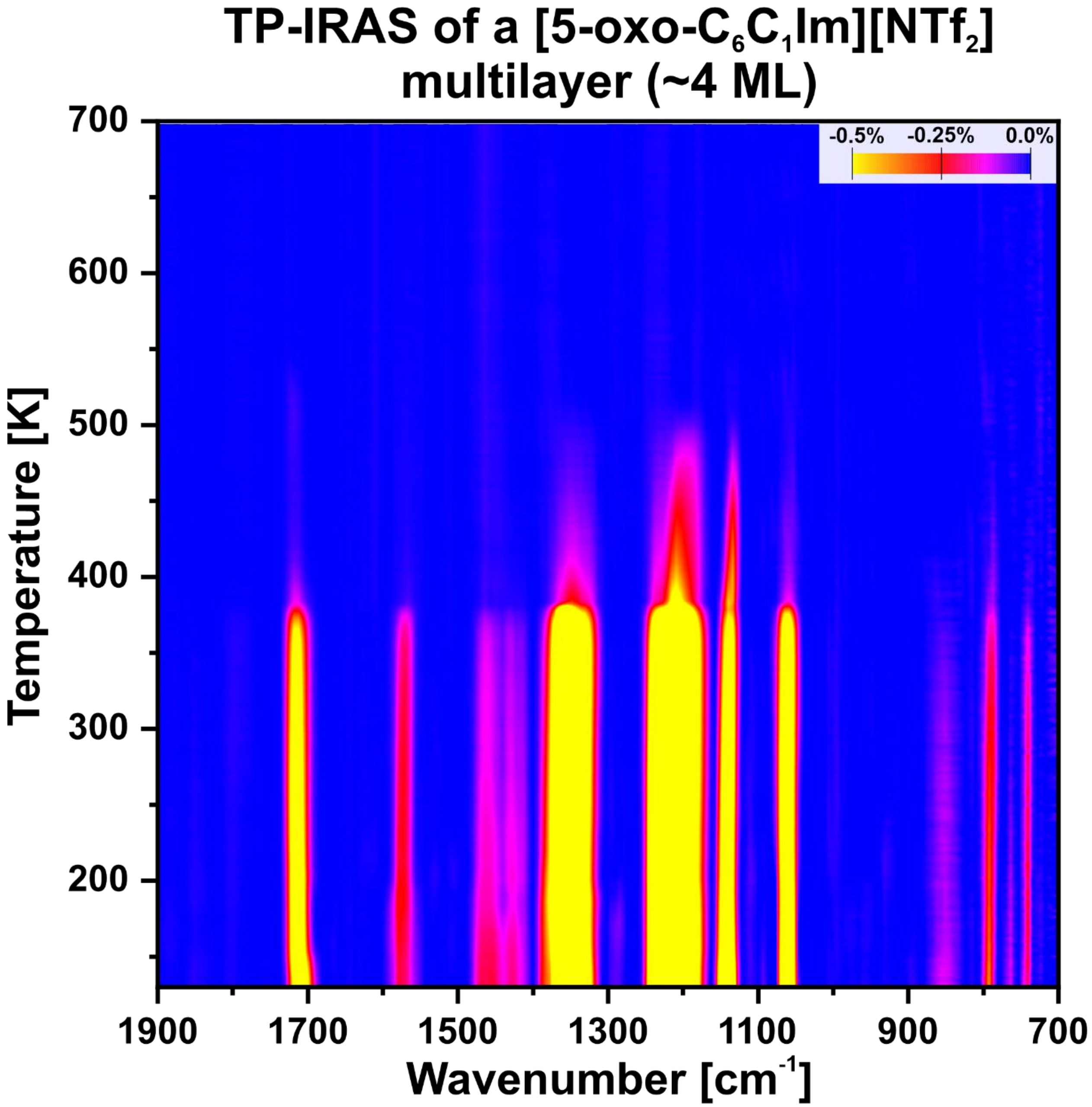


**Figure S2:** TP-IRAS of a [5-oxo-$C_6C_1$Im][$NTf_2$] multilayer from 130 to 700 K plotted as color plot.

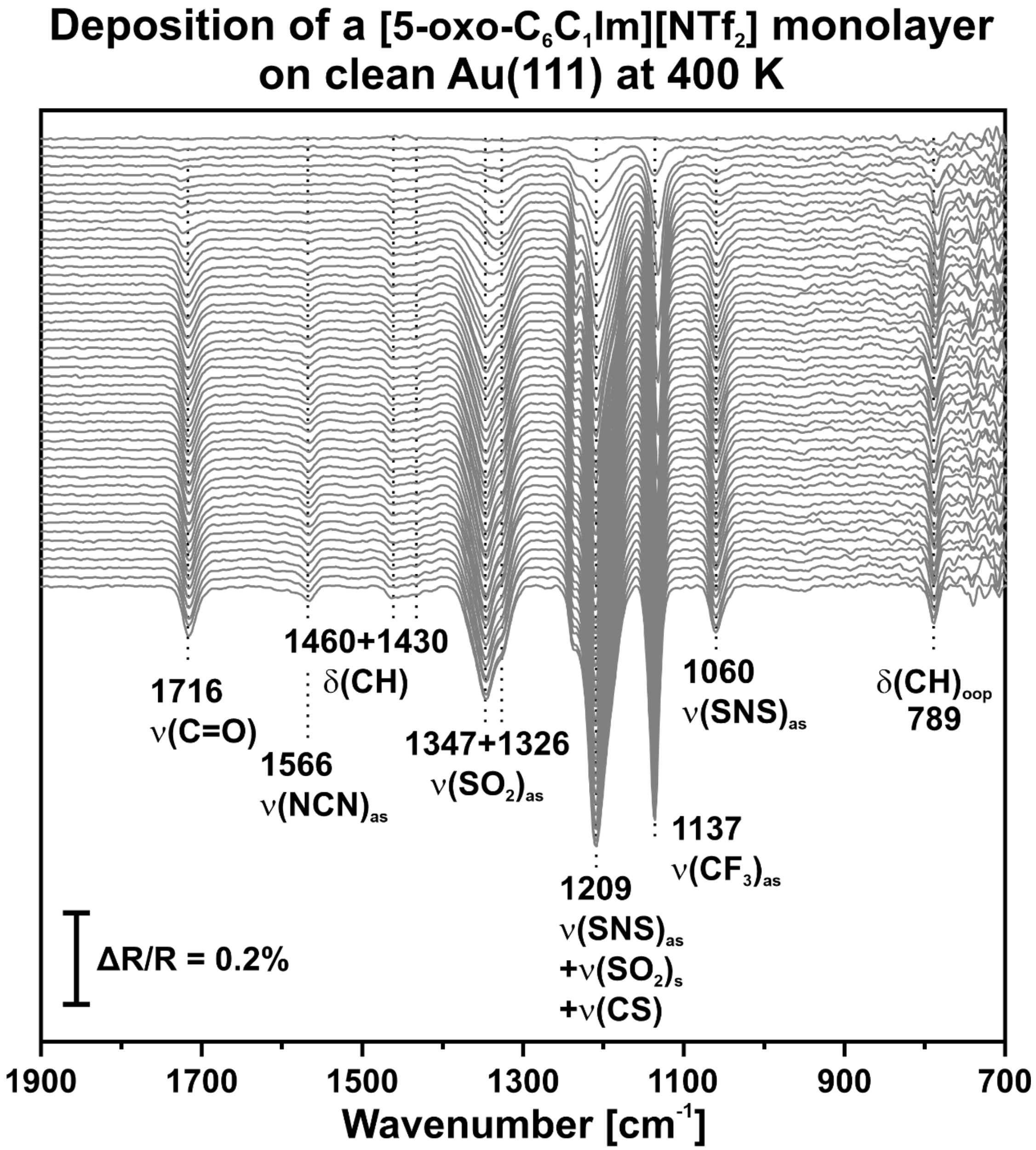


**Figure S3:** In-situ IRA spectra recorded during PVD of [5-oxo-$C_6C_1$Im][$NTf_2$] on Au(111) at 400 K. The spectra show the growth of a monolayer of the IL.

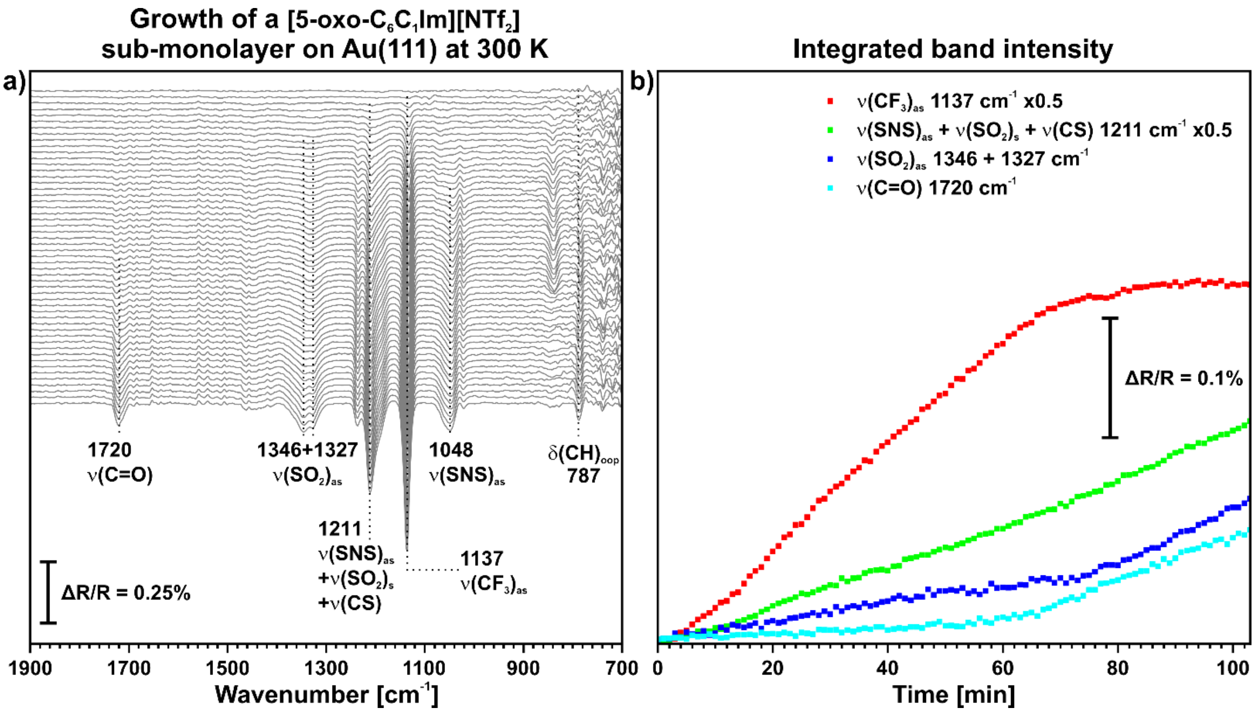


**Figure S4:** In-situ IRA spectra recorded during PVD of [5-oxo-$C_6C_1$Im][$NTf_2$] on Au(111) at 300 K. The spectra show the growth of a monolayer of the IL (a) as well as the integrated band intensity of the most prominent signals (b).

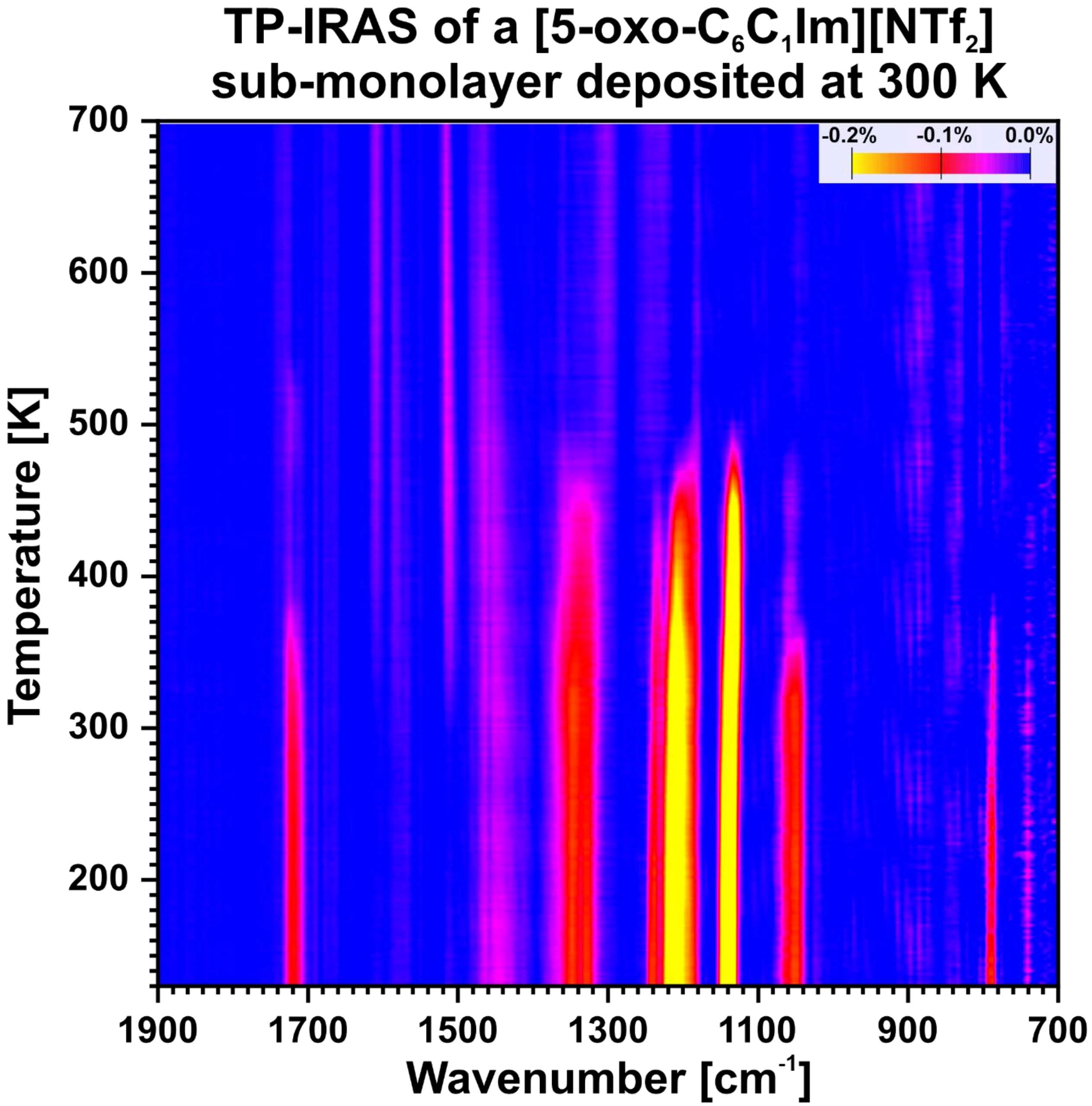


**Figure S5:** TP-IRAS of a [5-oxo-$C_6C_1$Im][$NTf_2$] sub-monolayer on Au(111) from 130 to 700 K, plotted as color plot.

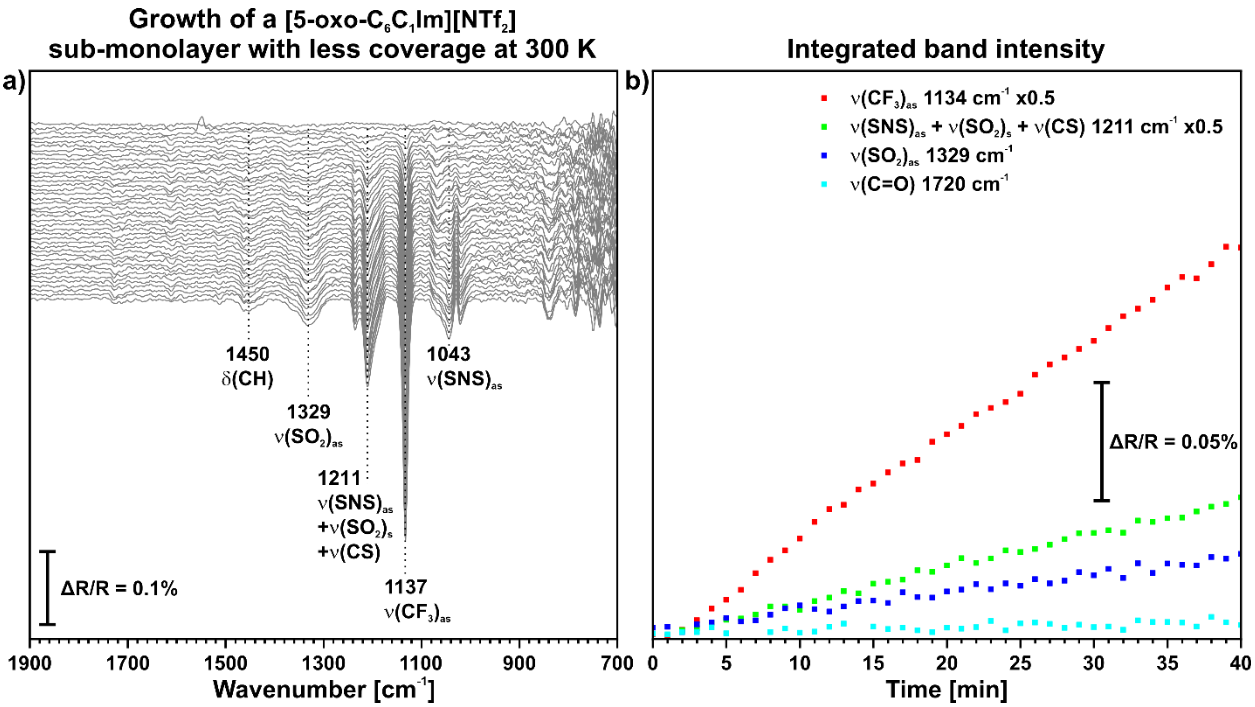


**Figure S6:** In-situ IRA spectra recorded during PVD of [5-oxo-$C_6C_1$Im][$NTf_2$] on Au(111) at 300 K with less coverage compared to Figure S4. The spectra show the growth of a monolayer of the IL (a) as well as the integrated band intensity of the most prominent signals (b).

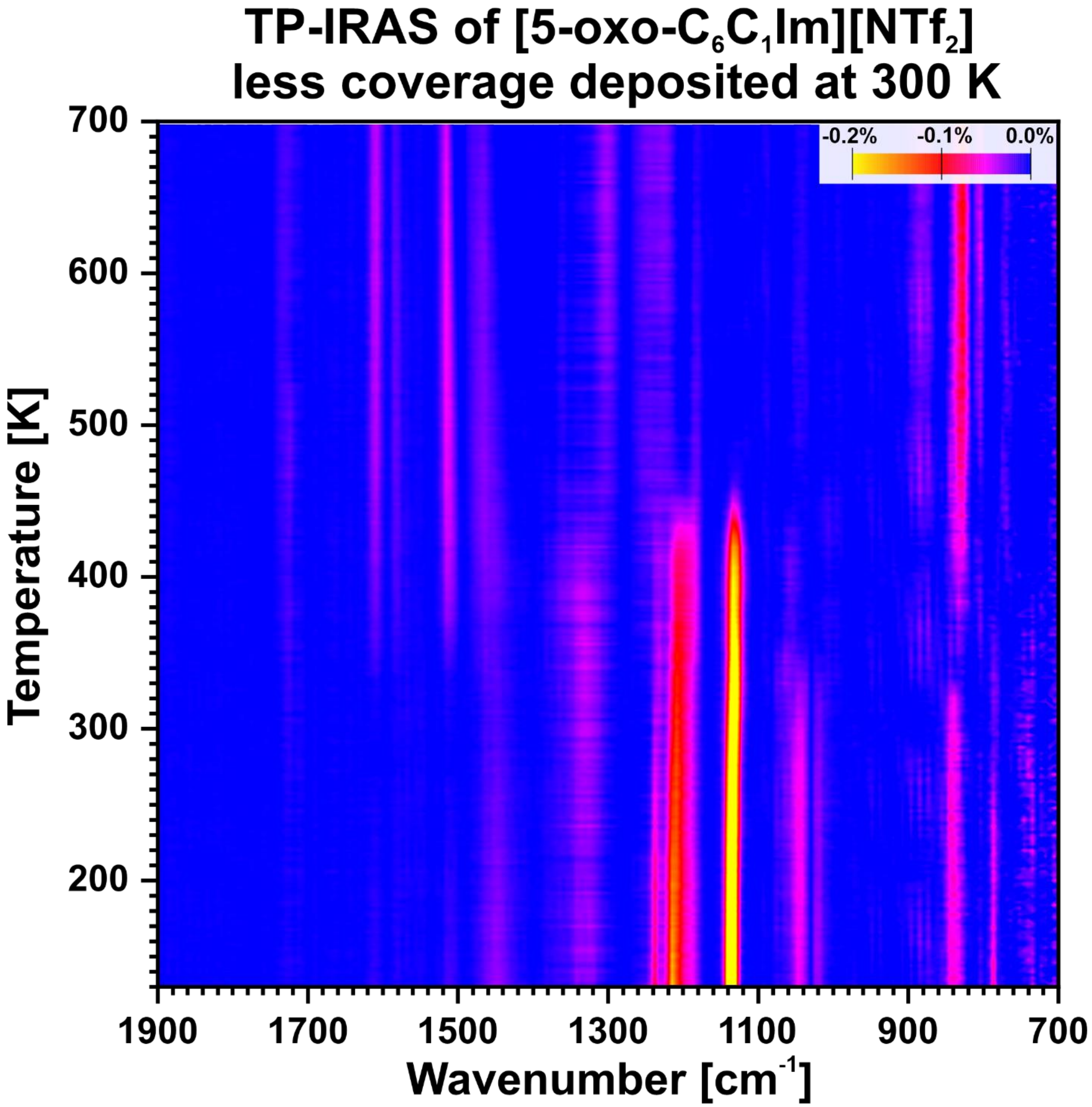


**Figure S7:** TP-IRAS of a [5-oxo-$C_6C_1$Im][$NTf_2$] sub-monolayer on Au(111) from 130 to 700 K, plotted as color plot.

**Table S1:** Detailed preparation and scanning parameter of all STM images.

| Figure | $T_{max}$ sample | $T_{prep}$ sample | $T_{STM}$ | $U_b$ | $I_T$ |
|---|---|---|---|---|---|
| 7a | 135 K | 135 K | 100 K | 1.6 V | 210 pA |
| 7b | 135 K | 135 K | 100 K | 1.6 V | 230 pA |
| 7c | 135 K | 135 K | 100 K | 1.6 V | 270 pA |
| 7d | 200 K | 120 K | 130 K | 1.6 V | 230 pA |
| 7e | 200 K | 120 K | 130 K | 1.6 V | 220 pA |
| 7f | 200 K | 120 K | 130 K | 1.6 V | 240 pA |
| 7g | 260 K | 130 K | 100 K | 1.6 V | 440 pA |
| 7h | 260 K | 130 K | 100 K | 1.6 V | 200 pA |
| 7i | 260 K | 130 K | 100 K | 1.6 V | 200 pA |

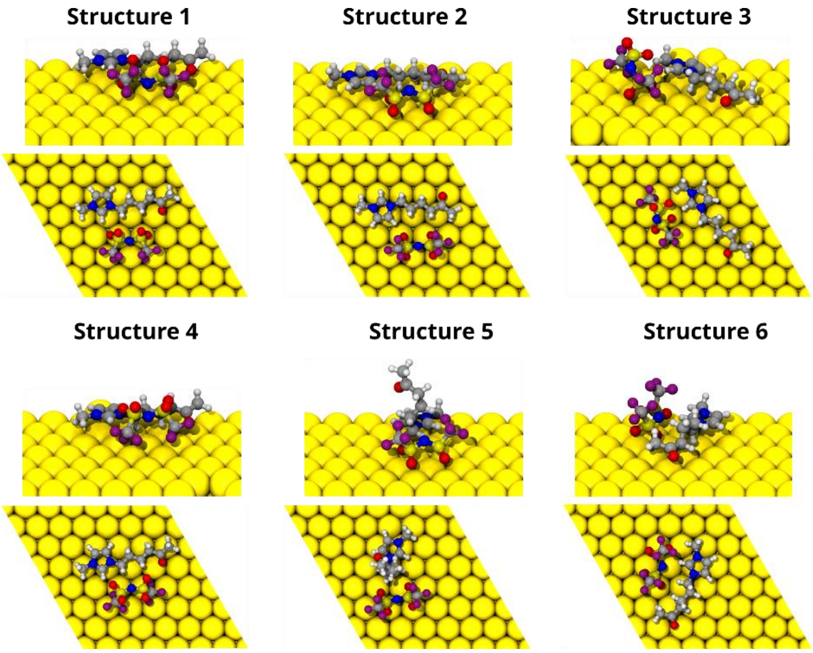


**Figure S8**: Six of the optimized [5-Oxo$C_1C_6$Im][$NTf_2$] dimer adsorption geometries on Au(111), with decreasing adsorption energy (see Table S2). The first three and the last one have the highest adsorption energy (or: lowest total energy) of all 20 structures, four and five are representative examples of other adsorption patterns with higher energies. Structure 6, whose initial geometry was taken from an abundant adsorption pattern occurring during the classical MD simulations, has the lowest total energy and is thus the most stable adsorption pattern, proving that the force field samplings gave stable structures in excellent agreement with DFT.

**Table S2**: Absolute energies of the different calculated species shown in Figure S8, as well as adsorption energies for the adsorbate species.

| **Species** | **Total energy (eV)** | **Adsorption energy (eV)** |
|---|---|---|
| 5-Oxo$C_1C_6$Im | -172.030402 | |
| $NTf_2$ | -87.726836 | |
| 7x7-Au(111) (clean) | -694.514632 | |
| Structure 1 | -958.359753 | 4.087883 |
| Structure 2 | -958.278453 | 4.006583 |
| Structure 3 | -958.226790 | 3.994920 |
| Structure 4 | -957.750108 | 3.478238 |
| Structure 5 | -957.790225 | 3.518355 |
| Structure 6 | -958.381636 | 4.109766 |

**[5-oxo-$C_6C_1$Im]$^+$ model**

The underlying Lennard-Jones parameters and partial charges are given in Table S3.

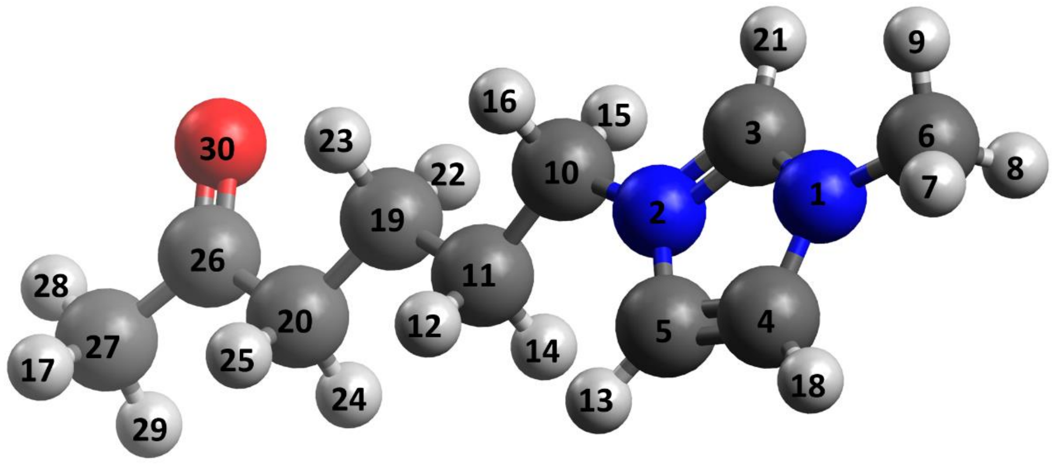


**Table S3**: Interaction parameters and partial charges of [5-oxo-$C_6C_1$Im]$^+$:

| Atom Number | Element | Partial Charge / e | ε / kcal/mol | σ /Å |
|---|---|---|---|---|
| 1 | N | +0.14215 | 0.204 | 3.201 |
| 2 | N | +0.16875 | 0.204 | 3.201 |
| 3 | C | -0.04744 | 0.099 | 3.315 |
| 4 | C | -0.16398 | 0.099 | 3.315 |
| 5 | C | -0.26503 | 0.099 | 3.315 |
| 6 | C | -0.30228 | 0.108 | 3.398 |
| 7 | H | +0.16203 | 0.021 | 2.422 |
| 8 | H | +0.16203 | 0.021 | 2.422 |
| 9 | H | +0.16203 | 0.021 | 2.422 |
| 10 | C | -0.27646 | 0.108 | 3.398 |
| 11 | C | +0.05715 | 0.108 | 3.398 |
| 12 | H | +0.02630 | 0.021 | 2.600 |
| 13 | H | +0.28414 | 0.016 | 2.536 |
| 14 | H | +0.02630 | 0.021 | 2.600 |
| 15 | H | +0.14513 | 0.021 | 2.422 |
| 16 | H | +0.14513 | 0.021 | 2.422 |
| 17 | H | +0.13712 | 0.021 | 2.600 |
| 18 | H | +0.24787 | 0.016 | 2.536 |
| 19 | C | +0.10722 | 0.108 | 3.398 |
| 20 | C | -0.36892 | 0.108 | 3.398 |
| 21 | H | +0.24744 | 0.016 | 2.447 |
| 22 | H | +0.02236 | 0.021 | 2.600 |
| 23 | H | +0.02236 | 0.021 | 2.600 |

| | | | | |
|---|---|---|---|---|
| 24 | H | +0.08603 | 0.021 | 2.600 |
| 25 | H | +0.08603 | 0.021 | 2.600 |
| 26 | C | +0.77981 | 0.099 | 3.315 |
| 27 | C | -0.49123 | 0.108 | 3.398 |
| 28 | H | +0.13712 | 0.021 | 2.600 |
| 29 | H | +0.13712 | 0.021 | 2.600 |
| 30 | O | -0.57627 | 0.146 | 3.048 |

**$[NTf_2]^-$ model**

The underlying Lennard-Jones parameters and partial charges are given in Table S4.

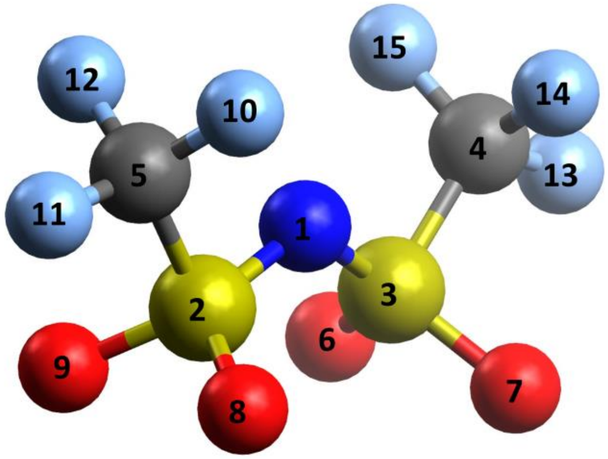


**Table S4**: Interaction parameters and partial charges of $[NTf_2]^-$:

| Atom Number | Element | Partial Charge / e | ε / kcal/mol | σ /Å |
|---|---|---|---|---|
| 1 | N | -0.75894 | 0.094 | 3.384 |
| 2 | S | +1.14184 | 0.282 | 3.532 |
| 3 | S | +1.14184 | 0.282 | 3.532 |
| 4 | C | +0.35524 | 0.108 | 3.398 |
| 5 | C | +0.35524 | 0.108 | 3.398 |
| 6 | O | -0.58754 | 0.146 | 3.048 |
| 7 | O | -0.58754 | 0.146 | 3.048 |
| 8 | O | -0.58754 | 0.146 | 3.048 |
| 9 | O | -0.58754 | 0.146 | 3.048 |
| 10 | F | -0.14751 | 0.083 | 3.034 |
| 11 | F | -0.14751 | 0.083 | 3.034 |
| 12 | F | -0.14751 | 0.083 | 3.034 |
| 13 | F | -0.14751 | 0.083 | 3.034 |
| 14 | F | -0.14751 | 0.083 | 3.034 |
| 15 | F | -0.14751 | 0.083 | 3.034 |
